# A folate metabolism-related prognostic signature for predicting survival in patients with hepatocellular carcinoma

**DOI:** 10.3389/fgene.2026.1822019

**Published:** 2026-10-28

**Authors:** Le Lin, Xiaoyu Chen, Jianghao Wu, Changyu Li, Ming Yu, Binbin Cai

**Affiliations:** 1 Department of Obstetrics, The First Affiliated Hospital of Wenzhou Medical University, Wenzhou, Zhejiang, China; 2 Department of Hepatobiliary and Pancreatic Surgery, The First Affiliated Hospital of Wenzhou Medical University, Wenzhou, Zhejiang, China; 3 The First Clinical Medical Institute of Wenzhou Medical University, Wenzhou, Zhejiang, China; 4 Department of Surgical Oncology, The Third Affiliated Hospital of Wenzhou Medical University (Ruian People’s Hospital), Wenzhou, Zhejiang, China

**Keywords:** elastic net cox, folate metabolism-related genes, GSS, hepatocellular carcinoma, prognostic model

## Abstract

**Background:**

Hepatocellular carcinoma (HCC) is highly heterogeneous, and reliable molecular prognostic biomarkers remain limited. Folate metabolism contributes to nucleotide synthesis, methyl-donor generation, and redox homeostasis, but the prognostic relevance of folate metabolism-related genes (FAMGs) in HCC remains unclear.

**Methods:**

Differential expression and functional enrichment analyses of FAMGs were performed in TCGA-LIHC. A prognostic model was developed using univariate Cox and Elastic Net Cox regression and evaluated by bootstrap resampling and external validation in LIRI-JP and GSE10141. SHapley Additive exPlanations (SHAP) was used for model interpretation. Immune characteristics and predicted drug responses were assessed using CIBERSORT, ssGSEA, IPS, TIDE, and oncoPredict. GSS, a candidate gene outside the model, was further examined by single-cell transcriptomic analysis and *in vitro* experiments.

**Results:**

A total of 974 differentially expressed FAMGs were identified, and 231 prognosis-associated candidates were screened to construct a 20-gene model. The model achieved a C-index of 0.767 (95% CI, 0.718–0.815) in TCGA-LIHC and an optimism-corrected C-index of 0.676 after 999 bootstrap resamples. EPO, MYCN, PPAT, PON1, and PRDX1 showed the highest global SHAP importance. External validation showed prognostic discrimination in LIRI-JP (C-index = 0.735) but limited performance in GSE10141 (C-index = 0.495). Risk groups differed in immune-related features and predicted drug sensitivity, whereas TIDE-predicted immune checkpoint inhibitor response rates were not significantly different. Although GSS was not included in the final model, higher GSS expression was associated with poorer prognosis. Single-cell analysis showed increased GSS expression and a higher proportion of GSS-positive cells in malignant hepatocytes. *In vitro*, GSS knockdown inhibited Hepatocellular carcinoma (HCC) cell proliferation, migration, and invasion, whereas overexpression produced the opposite effects.

**Conclusion:**

A 20-gene FAMG-related prognostic model was developed for HCC and showed variable performance across external cohorts, indicating that further validation is required. GSS may contribute to malignant phenotypes in HCC and warrants further mechanistic investigation.

## 1 Introduction

Hepatocellular carcinoma (HCC) accounts for over 90% of primary liver cancers, with rising global incidence and poor 5-year survival rates below 20% (Sung et al., 2021). Current treatment modalities for HCC include surgical resection, local ablation, transarterial chemoembolization, systemic targeted therapy, and immunotherapy (Anwanwan et al., 2020). However, the significant variability in treatment response among patients highlights the limitations of current anatomy-based staging systems such as TNM or BCLC: they fail to adequately capture the molecular heterogeneity of the disease, impeding precise guidance for individualized treatment strategies (Llovet et al., 2008). This situation underscores the urgent need for molecular tools capable of effectively predicting prognosis and guiding therapy.

However, HCC exhibits marked molecular and clinical heterogeneity, and even patients at similar clinical stages may show substantial differences in disease progression and treatment outcomes (Llovet et al., 2021). Therefore, on the basis of existing clinical staging and pathological indicators, it is of great importance to further explore molecular signatures that reflect the biological heterogeneity of HCC and facilitate prognostic risk stratification of patients.

Folate metabolism (FAM) is an integral component of the one-carbon metabolic network; by providing one-carbon units, it participates in purine and thymidylate synthesis, methyl-donor production, and maintenance of cellular redox homeostasis, thereby supporting cell proliferation and metabolic homeostasis (Yang et al., 2021; Quevedo et al., 2022). Abnormal FAM has been recognized to be closely associated with metabolic reprogramming of tumor cells, and in HCC, alterations in folate cycle-related enzymes and one-carbon metabolism may also participate in tumor initiation and progression (Quevedo et al., 2022). In addition, epidemiological studies have suggested that lower folate levels are associated with liver injury and an increased risk of HCC, providing population-level evidence for a potential link between folate status and hepatocarcinogenesis (Welzel et al., 2007; Persson et al., 2013). Previous studies have constructed various HCC prognostic signatures based on immune-related genes and broadly defined metabolism-related genes, indicating that transcriptomic molecular information can complement conventional clinical prognostic indicators from different biological dimensions (Zhou et al., 2022; Weng et al., 2021). In contrast, systematic and integrated studies on the expression abnormalities, prognostic associations, immune characteristics, and biological functions of candidate genes of folate metabolism-related genes (FAMGs) remain relatively limited. Therefore, systematically evaluating the relationships between FAMGs and HCC prognosis and tumor biological characteristics warrants further investigation.

On this basis, this study first identified differentially expressed FAMGs in HCC and analyzed their related biological functions; subsequently, a FAMGs-related prognostic risk model was constructed based on the TCGA-LIHC cohort, and the stability and generalizability of the model were evaluated through Bootstrap resampling and independent external cohorts, with SHAP analysis used to interpret the predictive contribution of model genes. Building on this, this study further compared immune cell composition, immune-related functions, and computationally predicted immunotherapy and drug response characteristics between risk groups. In addition, by integrating differential expression, prognostic associations, and known biological functions, this study selected GSS, a candidate gene outside the model, and further evaluated its expression characteristics and potential biological role in HCC by combining single-cell transcriptome analysis with *in vitro* functional experiments. Through the above analyses, this study aims to further understand the links between FAMGs-related molecular signatures and the prognostic heterogeneity and malignant phenotype of HCC at two levels: prognostic risk stratification and candidate gene functional investigation.

## 2 Materials and methods

### 2.1 Data download

The training set for prognostic model construction consisted of gene expression profiles (TPM) and clinical data for 371 HCC tumor samples and 50 normal controls obtained from The Cancer Genome Atlas (TCGA) (https://portal.gdc.cancer.gov/). After matching gene-expression profiles with clinical survival data, a total of 318 HCC patients with complete survival information were enrolled for prognostic analysis (108 death events). To validate the external generalization performance of the model, the GSE10141 cohort was retrieved from the Gene Expression Omnibus (GEO) database as an independent validation set, which consisted of 80 HCC samples whose gene-expression data were generated on the GPL5474 platform (Human 6k Transcriptionally Informative Gene Panel for DASL), of which 62 samples (21 death events) were annotated as available for prognostic analysis. RNA-seq gene expression data and corresponding clinical follow-up information of the LIRI-JP (Liver Cancer–RIKEN, Japan) cohort were obtained from the Pan-Cancer Analysis of Whole Genomes (PCAWG) data platform (https://pcawg.xenahubs.net) as a second independent external validation set. After matching gene expression data with clinical survival information and excluding cases with missing survival data, a total of 68 HCC patients were finally included, of whom 18 died, for independent external validation of the prognostic model. Additionally, the scRNA-seq dataset GSE149614 (10 HCC samples, 8 normal controls) was downloaded for single-cell level analysis.

### 2.2 Screening of FAMGs and analysis of differentially expressed genes

From the GeneCards database (https://www.genecards.org) (Safran et al., 2010), we identified 2,043 protein-coding FAMGs, each with a correlation score >1. Differential expression analysis was performed between tumors and normal controls in the TCGA-LIHC cohort: after log2(TPM+1) transformation of the expression matrix, testing was performed using the empirical Bayes (eBayes) method of the limma package (v3.60.4), and the Benjamini–Hochberg (BH) method was used to control the false discovery rate (FDR). The thresholds for significant difference were adjusted *p*adj < 0.05 and |log2FC| > 0.5. The screened differentially expressed FAMGs were designated as DE-FAMGs, and visualized by heatmap and volcano plot.

### 2.3 GO and KEGG enrichment analysis for DE-FAMGs

First, GO and KEGG enrichment analyses were performed using the clusterProfiler package (version 4.12.6) in R language (version 4.4.2) (Yu et al., 2012). The specific process included (Sung et al., 2021): in the data preprocessing stage, gene symbols were converted into Entrez IDs through the org.Hs.eg.db package (version 3.19.1) and invalid identifiers were filtered (Howe et al., 2021); (Anwanwan et al., 2020) GO analysis used enrichGO function with pvalueCutoff = 1 for genome-wide analysis, and then selected the significant entries with p.adjust < 0.05, covering three ontology categories: biological process (BP), cellular component (CC) and molecular function (MF) (Ashburner et al., 2000). (Llovet et al., 2008) enrichKEGG function was used for KEGG analysis, with “hsa” as the parameter (Kanehisa and Goto, 2000). (Llovet et al., 2021) Visualization: bar plots were used to present the GO and KEGG enrichment analysis results. The GO bar plots were colored according to the three ontology categories of biological process (BP), cellular component (CC), and molecular function (MF); the KEGG bar plots were colored according to six primary functional categories: Cellular Processes, Environmental Information Processing, Genetic Information Processing, Human Diseases, Metabolism, and Organismal Systems. In addition, the functional similarity between enriched terms was calculated based on the pairwise_termsim function, and a tree diagram was drawn using the treeplot function to display the functional associations and hierarchical clustering relationships among enriched terms.

### 2.4 Prognostic model construction and SHAP analysis

With the TCGA-LIHC cohort as the training set, FAMGs with valid expression data were included for prognostic analysis. Univariate Cox proportional hazards regression analysis was performed using the survival package (version 3.7-0) in R language (v4.4.2), and *P* values for multiple testing were corrected using the Benjamini–Hochberg (BH) method; prognosis-related genes were screened at an adjusted *P* value (*p*.adjust) < 0.05. Subsequently, Elastic Net Cox regression analysis was performed using the glmnet package, with the mixing parameter (α) and penalty parameter (λ) optimized through 10-fold cross-validation; model parameters were determined according to the cross-validation results, and genes with non-zero regression coefficients were retained to construct the prognostic risk model. The risk score of each patient was calculated from the Elastic Net Cox regression coefficient and the standardized expression value of each gene using the formula Risk score = Σ(β_i_ × X_i_), where β_i_ is the regression coefficient of the i-th gene and X_i_ is the standardized expression value of the corresponding gene. To compare the modeling performance of different variable selection methods, LASSO Cox regression analysis was further performed on the same prognosis-related candidate genes, and the penalty parameter was determined by cross-validation. The Harrell concordance index (C-index) was used to evaluate the discriminative ability of the Elastic Net Cox and LASSO Cox models.

To evaluate the internal stability and potential overfitting of the model, internal validation was performed using Bootstrap resampling, repeated 999 times in total. In each resampling, the complete model construction procedure was re-executed, including univariate Cox regression analysis, BH multiple-testing correction, Elastic Net Cox regression, and cross-validation parameter optimization. The C-index of the model obtained from each resampling was calculated in both the Bootstrap samples and the original training cohort; the average difference between the two was used to estimate the optimism, which was subtracted from the C-index of the original model to obtain the Bootstrap-corrected C-index. Meanwhile, the distribution of the number of variables retained by the model in each resampling and the selection frequency of each gene were summarized to evaluate the stability of variable selection. In addition, the number of endpoint events in the training cohort was counted, and the ratio of the number of candidate variables to the number of endpoint events, as well as the events per variable (EPV) of the final model, were calculated respectively. The EPV of the final model was defined as the ratio of the number of endpoint events in the training cohort to the number of predictors finally included in the model, and was used to describe the relationship between model complexity and the number of available events.

Finally, SHapley Additive exPlanations (SHAP) analysis was implemented to interpret the prognostic model and quantify the contribution of each gene to model predictions (Lundberg et al., 2017). Since the final risk-score model represented a linear combination of the expression levels of 20 genes, the SHAP value for each gene in each patient was calculated as SHAP_i = β_i × (x_i − x̄_i), where β_i denotes the corresponding Cox regression coefficient of gene i, x_i is the gene-expression value for the given patient, and x̄_i is the mean expression level of gene i across the TCGA-LIHC training cohort. The SHAP baseline value of the model E[f(x)] was defined as the mean risk score of the training cohort. Global SHAP importance was quantified by the mean absolute SHAP value (mean |SHAP|) of each gene across the 318 training patients to evaluate the overall contribution of each gene to cohort-level prediction. Local SHAP analysis was used to interpret the direction and magnitude of gene-specific contributions to predictions for individual patients, satisfying f(x) = E[f(x)]+ΣSHAP_i. All analyses were performed in R (v4.4.2). The shapviz package (version 0.9.3) was utilized to visualize SHAP importance bar plots, bee-swarm plots, waterfall plots, and force plots. The SHAP approach followed the method proposed by Lundberg and Lee (Lundberg et al., 2017).

### 2.5 Dimensionality reduction and risk stratification

To assess the discriminative power of the risk model, we conducted principal component analysis (PCA) using the prcomp function (Baca et al., 2021), followed by UMAP and t-SNE (perplexity = 10, max_iter = 500) to visualize high-dimensional gene expression data (Liu X. et al., 2021). The cutoff value for risk stratification was determined as the median risk score (−0.0858) of the TCGA-LIHC training cohort, and patients were accordingly divided into high-risk and low-risk groups. To avoid data-driven threshold optimization during validation, this cutoff value together with the regression coefficients of the model were kept fixed and directly applied to the two independent validation cohorts (GSE10141 and LIRI-JP). No re-estimation of the cutoff was performed in the validation cohorts.

### 2.6 Survival and prognostic analysis

Kaplan-Meier survival curves were generated using the survminer package (version 0.4.9), with log-rank tests applied to compare differences between high- and low-risk groups (Lu et al., 2022). Univariate and multivariate Cox regression analyses were performed to evaluate independent prognostic factors, and results were visualized via forest plots (hazard ratios [HR] with 95% confidence intervals) (Liu et al., 2019; Li et al., 2022). Kaplan-Meier survival analysis as well as univariate and multivariate Cox regression were pre-specified prognostic hypothesis-testing procedures, and two-sided nominal *p*-values were reported.

### 2.7 Model validation and clinical correlation analysis

The predictive performance of the prognostic model was evaluated in the TCGA-LIHC training cohort and in the two independent external validation cohorts GSE10141 and LIRI-JP. The Harrell concordance index (C-index) and its 95% confidence interval (95% CI) were used to evaluate the overall discriminative ability of the model. Time-dependent receiver operating characteristic (ROC) curves at 1, 3, and 5 years were plotted using the timeROC package (version 0.4) in R language (v4.4.2), and the time-dependent area under the curve (tdAUC) and its 95% CI were calculated at the corresponding time points to assess the prognostic discriminative ability of the model at different time points. Meanwhile, the consistency between the predicted risk and the actual survival outcomes was evaluated by the calibration slope and calibration curves. During external validation, the risk scores of the validation cohorts were calculated and risk stratification was performed using the data standardization parameters, model regression coefficients, and risk score cutoff value determined in the training cohort; the model was not refitted and the cutoff value was not re-optimized in the validation cohorts, and no joint standardization or batch effect correction was performed between the training cohort and the external validation cohorts.

The predictive accuracy of the risk model was assessed using time-dependent receiver operating characteristic (ROC) curves at 1, 3, and 5 years (timeROC package (version 0.4)) (Blanche et al., 2013). Based on the TCGA-LIHC training cohort, a nomogram integrating risk score and clinical variables was constructed using the rms package (version 8.1-1). Calibration curves and time-dependent ROC curves at 1-, 3-, and 5-year were employed to evaluate the calibration and discriminative performance of the nomogram. The ComplexHeatmap package was used to generate heatmaps illustrating the association between risk score and clinical characteristics. Associations between risk stratification and pre-specified clinical features were assessed by the χ^2^ test, and nominal *p*-values were reported. A *p* < 0.05 was regarded as the threshold for statistical significance.

### 2.8 Functional enrichment analysis of model genes

To further investigate the potential biological functions of the 20 model genes, Gene Ontology (GO) Biological Process (BP) enrichment analysis was performed using the clusterProfiler package. Multiple-testing correction was conducted using the Benjamini–Hochberg method, and an adjusted *P* value (*p*.adjust) < 0.05 was considered statistically significant. Representative biological processes were visualized based on the significantly enriched GO-BP terms.

### 2.9 Computational immune profiling approach

The study employed a comprehensive computational approach to evaluate tumor immune microenvironment characteristics in HCC patients. For immune cell infiltration analysis, we performed CIBERSORT deconvolution using the LM22 signature matrix with 1,000 permutations to estimate relative proportions of 22 immune cell types (Wang et al., 2020). Fitting metrics including the CIBERSORT permutation *p*-value, correlation coefficient, and root-mean-square error (RMSE) were simultaneously obtained for each sample. No additional sample-exclusion threshold was set according to the CIBERSORT permutation *p*-value in the present study. All samples with successfully completed deconvolution and available risk-stratification information were retained for inter-group comparison. Ultimately, 318 TCGA-LIHC tumor samples with risk-group assignments were included for differential immune-cell infiltration analysis. Immune-related functional enrichment was assessed through ssGSEA using an immune gene set collection (immune.gmt) (Fan et al., 2022). Statistical analyses included (Sung et al., 2021): Wilcoxon rank-sum tests for immune cell proportion comparisons between risk groups (Anwanwan et al., 2020), Chi-square tests for immune subtype distribution analysis, and (Llovet et al., 2008) Spearman correlation for functional pathway associations. Nominal *p*-values were reported for the above-mentioned analyses, and a *p* < 0.05 was defined as the threshold for statistical significance. All analyses were conducted in R (v4.4.2) with visualization using ggplot2 and ComplexHeatmap packages. Multiple testing correction was applied (FDR < 0.05) where appropriate.

### 2.10 Analysis of tumor immune microenvironment and drug response

To further characterize immune-related features and computationally predicted drug responses across different risk groups, patients in the TCGA-LIHC cohort were first classified into immune subtypes based on established immune-related molecular signatures. Immune subtype information was available for 311 patients, including 156 patients in the high-risk group and 155 patients in the low-risk group. Differences in immune subtype composition between the two risk groups were assessed using the chi-square (χ^2^) test. The Tumor Immune Dysfunction and Exclusion (TIDE) algorithm was used to evaluate immune evasion-related characteristics and the predicted response to immune checkpoint inhibitor therapy. Continuous TIDE-derived variables, including the overall TIDE score, T-cell dysfunction score, and T-cell exclusion score, were compared between the high- and low-risk groups using the Wilcoxon rank-sum test. In addition, the proportions of patients predicted by TIDE to be responders were compared between the two risk groups using Fisher’s exact test.

The immunophenoscore (IPS) was evaluated in all 318 tumor samples from the TCGA-LIHC training cohort using TCIA immunophenogram-related data/tools. The evaluated scores included the overall IPS, IPS-PD1, IPS-CTLA4, and IPS-PD1+CTLA4. Differences in these scores between the high- and low-risk groups were assessed using the Wilcoxon rank-sum test.

Drug sensitivity was predicted using the oncoPredict package with GDSC2 drug-response data as the training reference dataset, and cross-dataset adjustment was performed using an empirical Bayes approach. After obtaining predicted half-maximal inhibitory concentration (IC_50_) values for each drug in each patient, the distributions of predicted IC_50_ values were compared between the high- and low-risk groups using the Wilcoxon rank-sum test. The resulting *P* values from multiple drug comparisons were adjusted using the Benjamini–Hochberg method, and an FDR < 0.05 was considered statistically significant. All analyses were performed in R version 4.4.2, and the results were visualized using the RColorBrewer and ggplot2 packages.

### 2.11 scRNA-seq data quality control and processing

Seurat objects were constructed with the CreateSeuratObject function from the Seurat package (version 5.1.0) (Hao et al., 2021) (min.cells = 3, min.features = 200). The quality control procedure included the following criteria (Sung et al., 2021): genes expressed in at least three cells were retained (Anwanwan et al., 2020); cells with more than 200 detected genes and a total UMI count below 40,000 were included; and (Llovet et al., 2008) cells with mitochondrial gene content below 5% were kept. Subsequently, data underwent normalization with the NormalizeData function and scaling with the ScaleData function. After dimensionality reduction by PCA (20 components retained), the Harmony algorithm corrected for batch effects. A k-nearest neighbor graph was constructed with the FindNeighbors function. Cells were clustered with the FindClusters function, with results visualized using RunTSNE. Cell type annotation was carried out based on canonical markers referenced from the SingleR package (version 2.8.0) (Aran et al., 2019) and the CellMarker 2.0 database (http://117.50.127.228/CellMarker/).

### 2.12 Differential expression and survival analysis

To compare GSS expression between normal control and tumor samples, a bubble plot was generated from gene expression data using the ggpubr package (version 0.6.0) (ggpubr). Patients in the TCGA-LIHC cohort were divided into high- and low-expression groups based on the median GSS expression level. Kaplan-Meier survival analysis was performed with the survival package to assess the relationship between GSS expression and prognosis.

### 2.13 Identification of malignant cells

Copy-number variations (CNVs) at the cellular level were inferred with the inferCNV package (version 1.24.0) using the quality-controlled raw gene-count matrix. Chromosome and genomic start-position annotations for genes were retrieved via the AnnoProbe package (version 0.1.0). Genes located on mitochondrial and sex chromosomes (chrM, chrX, and chrY) were removed, and the remaining genes were sorted according to their genomic coordinates. Annotated non-malignant normal reference cells were designated as the reference group (ref_group_names = “normal”) to define the baseline copy-number status. The parameters for inferCNV were set as follows: cutoff = 0.1, cluster_by_groups = TRUE, hclust_method = “ward.D2”, analysis_mode = “samples”, denoise = TRUE, HMM = FALSE, plot_steps = FALSE, leiden_resolution = 0, and num_threads = 16. Median filtering and smoothing were subsequently applied to the inferred CNV signals using apply_median_filtering.

Unsupervised clustering of all cells using the K-means algorithm, based on total copy number variation (CNV) scores from inferCNV analysis, partitioned them into high- and low-CNV groups. Comparison of mean CNV scores identified the high-score cluster as malignant cells and the low-score cluster as non-malignant cells. To validate the reliability of this classification, we examined the distribution of malignant cells across tumor samples and normal control.

### 2.14 Cell culture

Human normal hepatic epithelial cell line THLE-2 (Delf-16595) and HCC cell line Huh-7 (Delf-10199) were obtained from WANWUSHENGWU (China). Human HCC cell lines Hep3B (CL-0102) and SNU-182 (CL-0610) were obtained from Procell (Wuhan, China). All cells were cultured under standard conditions (37 °C, 5% CO_2_) in respective media: THLE-2 in BEGM kit medium (CC-3170, Lonza Bioscience, Walkersville, United States), Huh-7 in DMEM (11965092, Gibco, Grand Island, United States) supplemented with 10% FBS (16600082, Gibco, United States) and 1% penicillin-streptomycin (C0222, Beyotime, Shanghai, China), Hep3B in Hep 3B2.1-7 Cell Complete Medium (CM-0102, Procell, Wuhan, China), and SNU-182 in SNU-182 Cell Complete Medium (CM-0610, Procell, China).

### 2.15 Cell transfection

Huh-7 cells were transfected with si-GSS (RiboBio, China) and its negative control (si-NC) (RiboBio, Guangzhou, China) with Lipofectamine RNAiMAX transfection reagent (13778100, Invitrogen, Carlsbad, United States) for 24 h before subsequent experiments. SNU-182 cells were transfected with oe-GSS (RiboBio, China) and oe-NC (RiboBio, China) plasmids using Lipofectamine™ 3000 transfection reagent (L3000075, Invitrogen, United States).

### 2.16 qRT-PCR

Total RNA extracted from cells with Trizol reagent (15596018CN, Thermo Fisher Scientific, Waltham, United States) was quantified for RNA concentration and purity with a NanoDrop spectrophotometer. cDNA was synthesized with HiScript III RT SuperMix (R323-01, Vazyme, Nanjing, China) and amplified on a Step-One Plus real-time PCR system with the SYBR Green qPCR kit (CL132-01, Vazyme, China) (primer sequences in Table 1).

**TABLE 1:** qRT-PCR primers.

Gene	Forward primer (5′-3′)	Reverse primer (5′-3′)
GSS	GAA​AGG​CGA​ACT​AGT​GTT​GGG	AGA​GCG​TGA​ATG​GGG​CAT​AG
GAPDH	AAT​GGG​CAG​CCG​TTA​GGA​AA	GCC​CAA​TAC​GAC​CAA​ATC​AGA​G

### 2.17 Western blot (WB)

Proteins extracted from cells using RIPA buffer (P0013B, Beyotime, China) were quantified with a BCA assay kit (P0009, Beyotime, China), separated by SDS-PAGE, and transferred onto PVDF membranes. Membranes blocked with 5% skim milk (2 h, room temperature) were incubated overnight at 4 °C with primary antibodies against GSS (1:1,000, Rabbit, 15712-1-AP, Proteintech, China) and GAPDH (1:8,000, Mouse, 60004-1-Ig, Proteintech, Wuhan, China), washed three times with TBST, and then incubated with HRP-conjugated secondary antibodies for 2 h at room temperature (Goat anti-Rabbit IgG, 1:8,000, 111-035-003, Jackson, ImmunoResearch, West Grove, United States; Goat anti-Mouse IgG, 1:8,000, 115-035-003, Jackson, United States). Protein bands were visualized using an ECL kit (34577, Thermo Fisher, United States) and analyzed for expression levels.

### 2.18 CCK-8 assay

Cells treated with various methods were cultured in 96-well plates (2 × 10^3^ cells/well) at 37 °C with 5% CO_2_ and assessed for proliferation at 0, 24, 48, and 72 h by adding 10 μL CCK-8 reagent (C0037, Beyotime) per well, reacting for 2 h, and measuring absorbance at 450 nm using a microplate reader.

### 2.19 EdU staining

Cell proliferation was evaluated using the BeyoClick™ EdU Cell Proliferation Kit (C0071S, Beyotime, China). Cells treated with various methods were cultured on circular coverslips in 12-well plates (1 × 10^4^ cells/well) for 12 h before EdU labeling (2 h), 4% paraformaldehyde fixation (15 min), 0.3% Triton X-100 permeabilization (15 min). After incubation with Click Reaction Buffer in the dark (30 min, room temperature), nuclei were counterstained with Hoechst 33342 (10 min).

### 2.20 Transwell migration/invasion assay

Cells treated with various methods (2 × 10^4^) suspended in 200 μL serum-free DMEM or RPMI-1640 were seeded into the upper chambers of a Transwell system, either pre-coated (for invasion assay) or uncoated (for migration assay) with Matrigel (354230, Corning, Corning, United States); the lower chambers contained 600 μL complete medium with serum. Following 24 h incubation at 37 °C with 5% CO_2_, migrated or invaded cells were fixed with 4% paraformaldehyde, stained with crystal violet for 2 min, and counted in five randomly selected fields per well under an inverted microscope.

### 2.21 Data analysis and statistical methods

Unless otherwise specified, all statistical tests were two-sided, and a *p* < 0.05 was set as the threshold for statistical significance. For exploratory analyses involving simultaneous testing of multiple hypotheses, multiple-comparison correction was performed according to specific analytical purposes. For differential expression analysis of FAMGs, the BH method was used to control the FDR (*p*adj < 0.05 and |log2FC| > 0.5). The BH method was applied for GO and KEGG functional enrichment analyses, with *p*.adjust < 0.05 defined as the significance cutoff. Nominal *p*-values were reported for pre-specified hypothesis tests, including Kaplan-Meier survival analysis, Cox regression, and association analyses of clinical features.

All *in vitro* quantitative experiments were performed with no fewer than three independent biological replicates (n ≥ 3), and data were presented as mean ± SEM. Statistical significance was assessed using Student’s t-test for two-group comparisons and two-way ANOVA for multi-timepoint analyses, performed with Microsoft Excel and GraphPad Prism 8.0. Significance levels are denoted as: ns, *p* > 0.05; *, *p* < 0.05; **, *p* < 0.01; ***, *p* < 0.001; ****, *p* < 0.0001.

## 3 Results

### 3.1 Identifying DE-FAMGs in HCC

A total of 2,043 FAMGs were obtained from the GeneCards database. In the TCGA-LIHC cohort, 974 DE-FAMGs were identified after limma eBayes testing with BH correction (padj < 0.05 and |log2FC| > 0.5), including 753 upregulated and 221 downregulated genes (Supplementary Table S1). Volcano plot was used to visualize the global differential-expression pattern of DE-FAMGs, and a heatmap was generated to display the top 10 most significantly differentially expressed DE-FAMGs (Top 10 DE-FAMGs) (Figures 1A,B).

**FIGURE 1:**
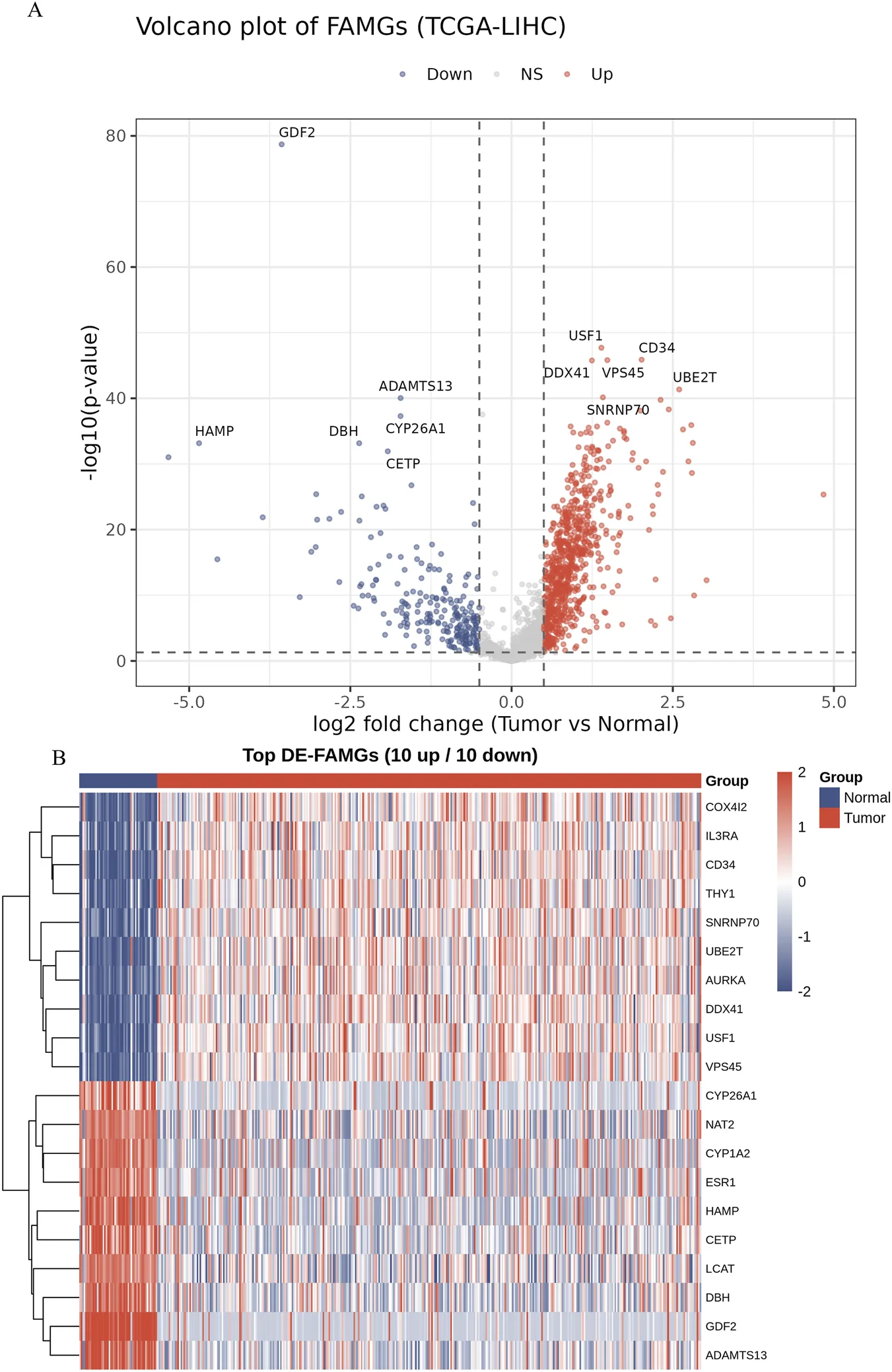
Identification and expression characteristics of differentially expressed folate metabolism-related genes (DE-FAMGs) in HCC. **(A)** Volcano plot of DE-FAMGs between HCC and normal liver tissues. **(B)** Heatmap of the expression of the top 10 most significantly differentially expressed DE-FAMGs between HCC and normal liver tissues. DE-FAMGs, differentially expressed folate metabolism-related genes; HCC, hepatocellular carcinoma.

### 3.2 Functional and pathway enrichment analysis of DE-FAMGs

To explore the potential biological functions of DE-FAMGs, GO and KEGG enrichment analyses were performed on the 974 DE-FAMGs. GO analysis showed that, in terms of biological process (BP), DE-FAMGs were mainly enriched in DNA replication, DNA recombination, DNA double-strand break repair, telomere organization and maintenance, and responses to exogenous compounds, oxidative stress, and hypoxia; in terms of cellular component (CC), they mainly involved chromosomal region, telomeric region, replication fork, focal adhesion, and cytosolic ribosome; and in terms of molecular function (MF), they mainly involved DNA-binding transcription factor binding, DNA catalytic activity, single-stranded DNA binding, and damaged DNA binding (Figures 2A,B). KEGG enrichment analysis showed that DE-FAMGs were mainly involved in pathways related to human diseases, cellular processes, genetic information processing, and metabolism, including hepatocellular carcinoma, the cell cycle, the HIF-1 signaling pathway, the p53 signaling pathway, homologous recombination, and DNA repair-related pathways. In addition, pathways such as one carbon pool by folate, folate transport and metabolism, antifolate resistance, and porphyrin metabolism were also enriched (Figures 2C,D). These results suggest that DE-FAMGs may be closely associated with HCC-related DNA replication and repair, cell cycle regulation, stress response, and metabolic processes, providing a basis for further investigation of their potential biological roles.

**FIGURE 2:**
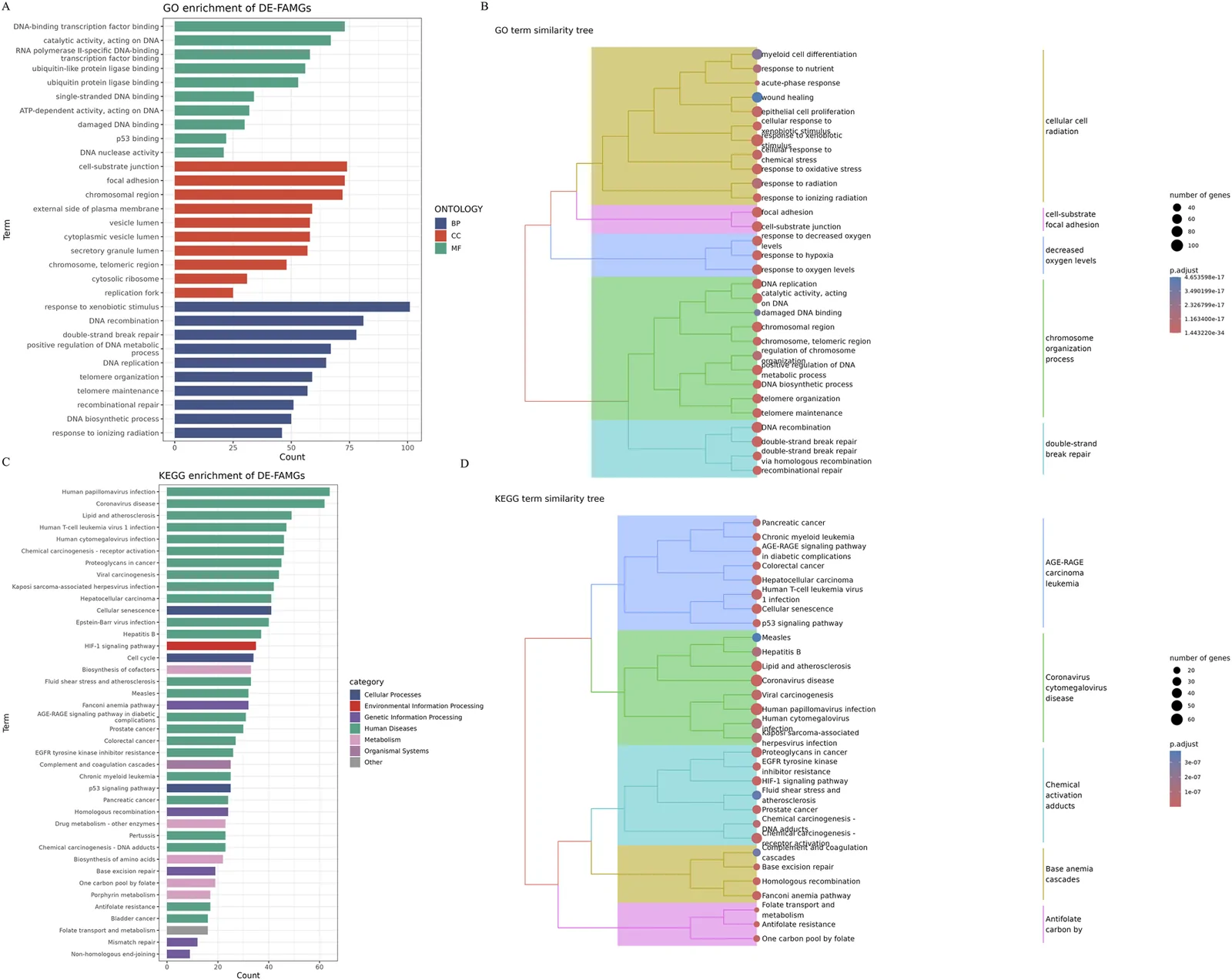
GO and KEGG functional enrichment analysis of 974 differentially expressed folate metabolism-related genes (DE-FAMGs) in HCC. **(A)** Bar plot of Gene Ontology (GO) enrichment analysis of DE-FAMGs, showing the top 10 significantly enriched terms in biological process (BP), cellular component (CC), and molecular function (MF), respectively. **(B)** Hierarchical clustering tree diagram of GO enriched terms based on functional similarity. **(C)** Bar plot of Kyoto Encyclopedia of Genes and Genomes (KEGG) pathway enrichment analysis of DE-FAMGs, showing the top 40 significantly enriched pathways colored according to KEGG primary functional categories. **(D)** Hierarchical clustering tree diagram of KEGG enriched pathways based on functional similarity. DE-FAMGs, differentially expressed folate metabolism-related genes; GO, Gene Ontology; BP, biological process; CC, cellular component; MF, molecular function; KEGG, Kyoto Encyclopedia of Genes and Genomes.

### 3.3 Prognostic risk model and SHAP interpretation for HCC

Based on the TCGA-LIHC training cohort, univariate Cox regression analysis was performed on FAMGs, and a total of 231 genes significantly associated with the prognosis of HCC patients were screened after Benjamini–Hochberg (BH) correction (adjusted *P* < 0.05, Figure 3A; Supplementary Table S2). Elastic Net Cox regression analysis was then performed, and the optimal parameters (α = 0.9, λ.min = 0.056) were determined through 10-fold cross-validation; finally, 20 genes were selected to construct the prognostic risk model (Figures 3B,C). Among them, the regression coefficients of PON1, LCAT, and SERPINF2 were negative, while those of the remaining 17 genes were positive. The risk score was calculated from the standardized expression value of each gene and the corresponding regression coefficient, with the specific formula as follows:

**FIGURE 3:**
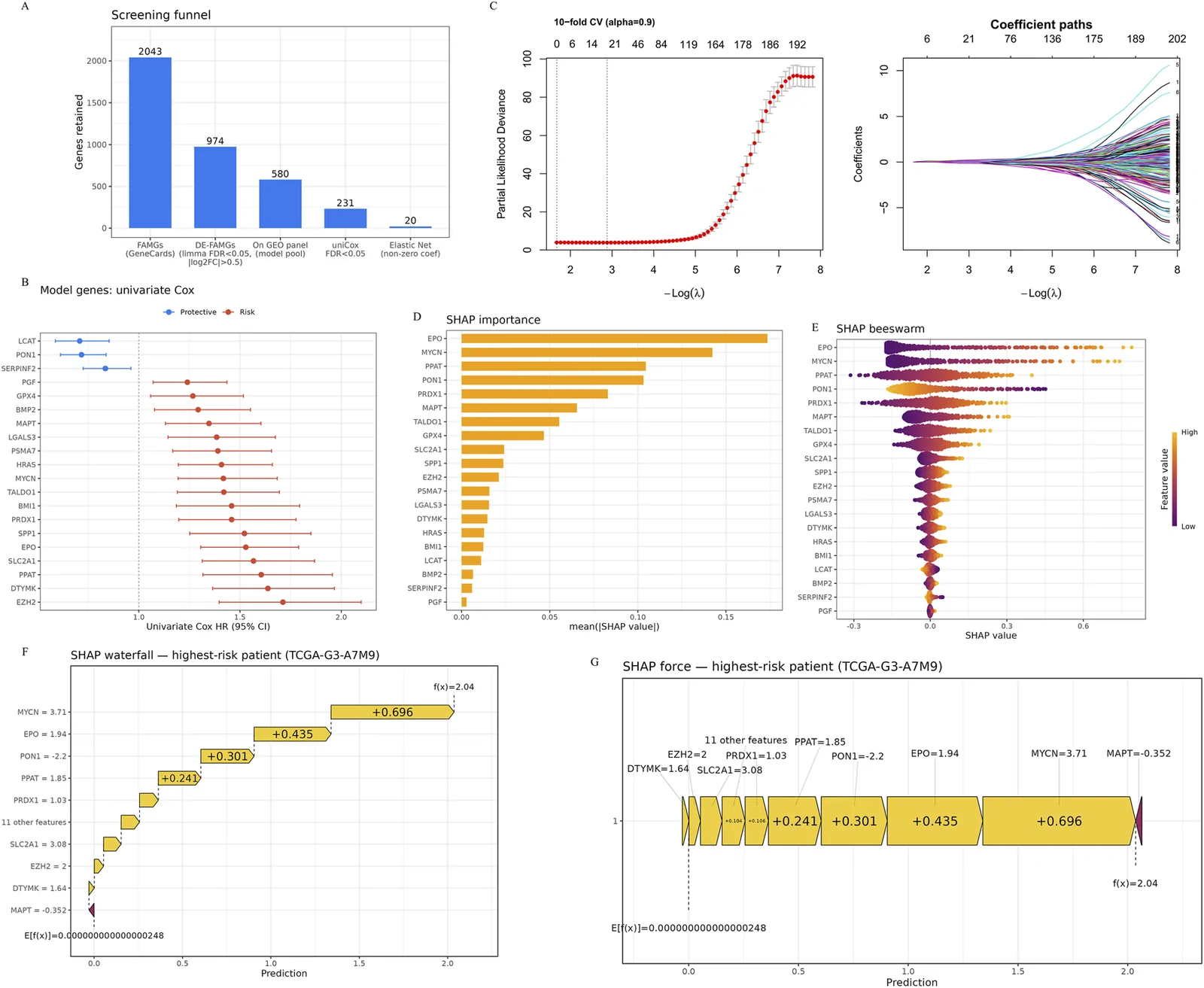
Screening of prognosis-related FAMGs, construction of the Elastic Net Cox model, and SHAP interpretation in HCC. **(A)** Funnel plot of univariate Cox regression analysis for screening prognosis-related candidate genes, in which a total of 231 candidate genes with statistical significance after FDR correction were identified. **(B)** Forest plot of univariate Cox regression for the final 20 model genes, showing the hazard ratio (HR) and 95% confidence interval of each gene. **(C)** Cross-validation curve and regression coefficient path of Elastic Net Cox regression, used to determine the optimal penalty parameters and screen model genes. **(D)** Global SHAP importance ranking of the 20 model genes, measured by the mean absolute SHAP value to quantify the overall contribution of each gene to model prediction. **(E)** SHAP beeswarm plot, showing the relationship between the expression level of each gene and its SHAP value and the influence on the direction of model prediction. **(F)** SHAP waterfall plot of a representative patient, showing the magnitude and direction of the contribution of each gene to the individual risk score. **(G)** SHAP force plot of the same representative patient, showing the positive or negative contributions of each model gene to the predicted risk. FDR, false discovery rate; HR, hazard ratio; SHAP, SHapley Additive exPlanations.

Risk score = 0.223 × EPO + 0.188×MYCN + 0.130 × PPAT + 0.104 × PRDX1 + 0.082 × MAPT + 0.070 × TALDO1 + 0.058 × GPX4 + 0.032 × SLC2A1 + 0.028 × SPP1 + 0.026 × EZH2 + 0.021 × PSMA7 + 0.018 × DTYMK + 0.019 × LGALS3 + 0.016 × HRAS + 0.015 × BMI1 + 0.008 × BMP2 + 0.004 × PGF −0.137 × PON1 − 0.014 × LCAT − 0.009 × SERPINF2.

The C-index of the model in the training cohort was 0.767 (95% CI: 0.718–0.815). For comparison, LASSO Cox regression selected the same 20 genes on the same candidate set, with a C-index of 0.766, indicating that the two methods had similar discriminative performance and suggesting that the signature is robust to the penalty method (Supplementary Figure S1A).

To evaluate the internal stability of the model, 999 Bootstrap resampling iterations were performed, and the complete variable selection and modeling procedure was repeated in each iteration. The results showed that the original C-index of the model was 0.767, the Bootstrap-corrected C-index was 0.676, the estimated optimism was 0.090, and the median number of variables retained by the resampled models was 37. The corrected discrimination remained acceptable and honestly quantified the optimism introduced by variable selection. A total of 108 endpoint events occurred in the training cohort; the events per variable (EPV) of the final 20-gene model was 5.4, while the events-to-variables ratio corresponding to the 231 candidate genes was 0.47, suggesting that the model still carries a certain risk of overfitting.

SHAP analysis showed that EPO, MYCN, PPAT, PON1, and PRDX1 made relatively large contributions to model prediction, with mean absolute SHAP values of 0.173, 0.142, 0.105, 0.103, and 0.083, respectively (Figure 3D). The beeswarm plot showed that increased expression of EPO, MYCN, and PRDX1 mainly corresponded to positive SHAP values, whereas increased expression of PON1 mainly corresponded to negative SHAP values (Figure 3E). At the individual level, patient TCGA-G3-A7M9, who had the highest risk score in the TCGA-LIHC training cohort, was selected for local SHAP analysis. The predicted risk score of this patient was 2.04. The SHAP waterfall plot and force plot showed that MYCN (SHAP value = +0.696), EPO (+0.435), PON1 (+0.301), and PPAT (+0.241) were the main contributing genes driving the elevated risk score of this patient (Figures 3F,G). Among them, the lower expression level of PON1 corresponded to a positive SHAP contribution, consistent with its negative regression coefficient in the model. These results indicate that EPO had the highest global SHAP importance in the training cohort, whereas MYCN contributed most to the individual risk prediction of this patient, reflecting the differences in the predictive contributions of different genes at the cohort level and the individual level. In addition, GO biological process enrichment analysis of the 20 model genes showed that these genes were mainly enriched in biological processes such as cellular response to chemical stress, positive regulation of MAPK and ERK1/ERK2 signaling cascades, regulation of kinase activity, and regulation of miRNA metabolic process (Supplementary Figure S1B).

### 3.4 Prognostic stratification and validation of the prognostic risk model

Dimensionality-reduction analyses, including PCA, UMAP, and t-SNE, showed distinct distribution patterns between the high- and low-risk groups (Figures 4A–C). Kaplan–Meier survival analysis showed that patients in the high-risk group had significantly poorer overall survival than those in the low-risk group in the TCGA-LIHC training cohort and the LIRI-JP external validation cohort. In contrast, the survival difference between the high- and low-risk groups did not reach statistical significance in the GSE10141 cohort (log-rank *P* = 0.093), and the model yielded a C-index of 0.495 in this cohort (Figures 5A–C). These findings indicate that the model showed prognostic stratification in TCGA-LIHC and LIRI-JP but limited performance in GSE10141, suggesting that its cross-platform generalizability requires further validation. Notably, GSE10141 used a DASL 6K gene expression profiling platform based on formalin-fixed paraffin-embedded (FFPE) samples, and no death events were observed in this cohort during the first year of follow-up. In contrast, LIRI-JP used an RNA-seq sequencing platform, which is closer to the detection technology of the TCGA-LIHC training cohort. These results indicate that the model has certain prognostic stratification ability in the TCGA-LIHC and LIRI-JP cohorts but limited predictive performance in the GSE10141 cohort, suggesting that its cross-platform generalizability still requires further validation.

**FIGURE 4:**
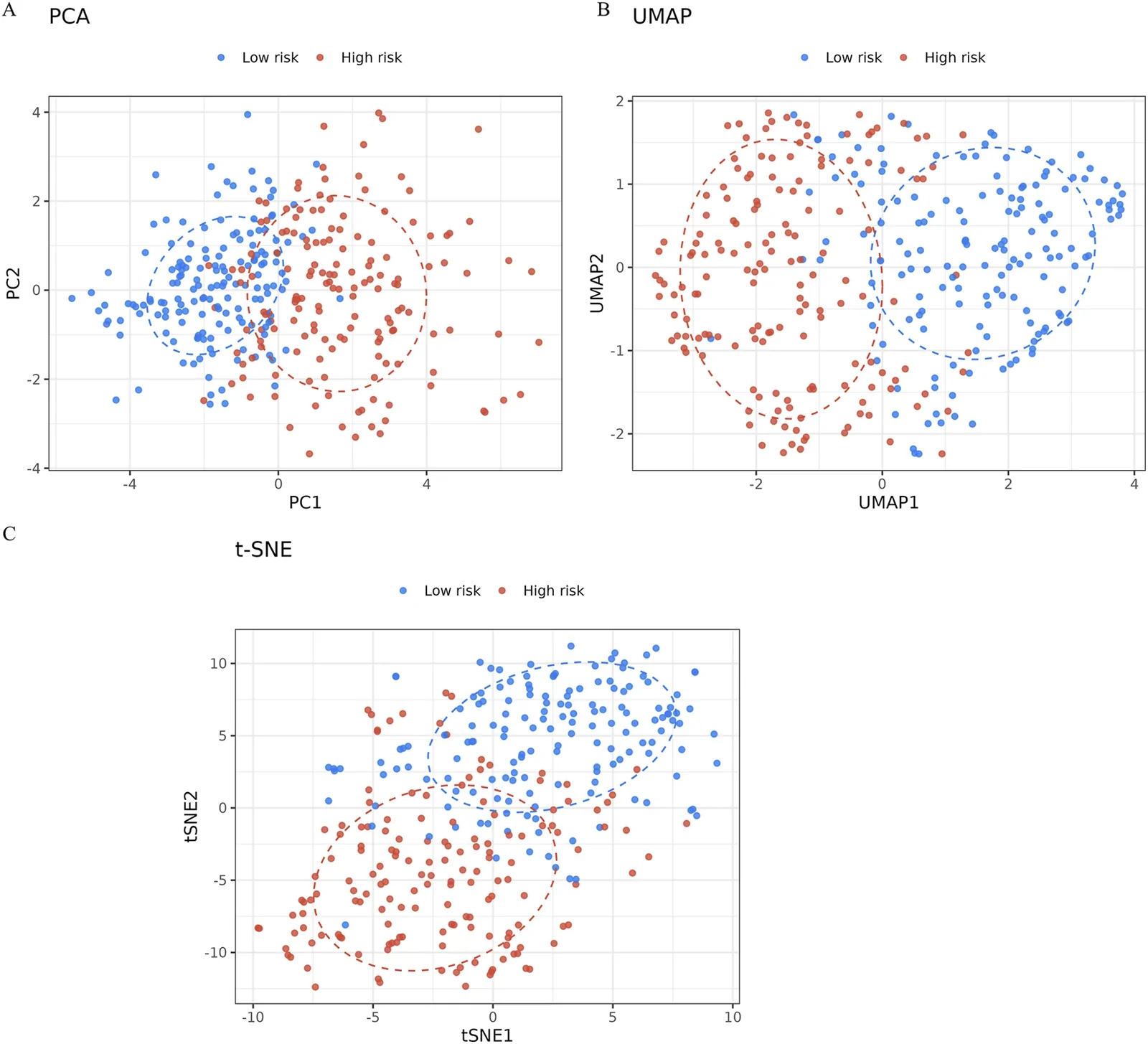
Dimensionality reduction analysis of patients in the high- and low-risk groups of the 20-gene prognostic model. **(A)** Principal component analysis (PCA). **(B)** Uniform manifold approximation and projection (UMAP) analysis. **(C)** t-distributed stochastic neighbor embedding (t-SNE) analysis. Different colors represent patients in the high-risk and low-risk groups, respectively, visualizing the distribution of the two groups of patients in the feature space of model gene expression. PCA, principal component analysis; UMAP, uniform manifold approximation and projection; t-SNE, t-distributed stochastic neighbor embedding.

**FIGURE 5:**
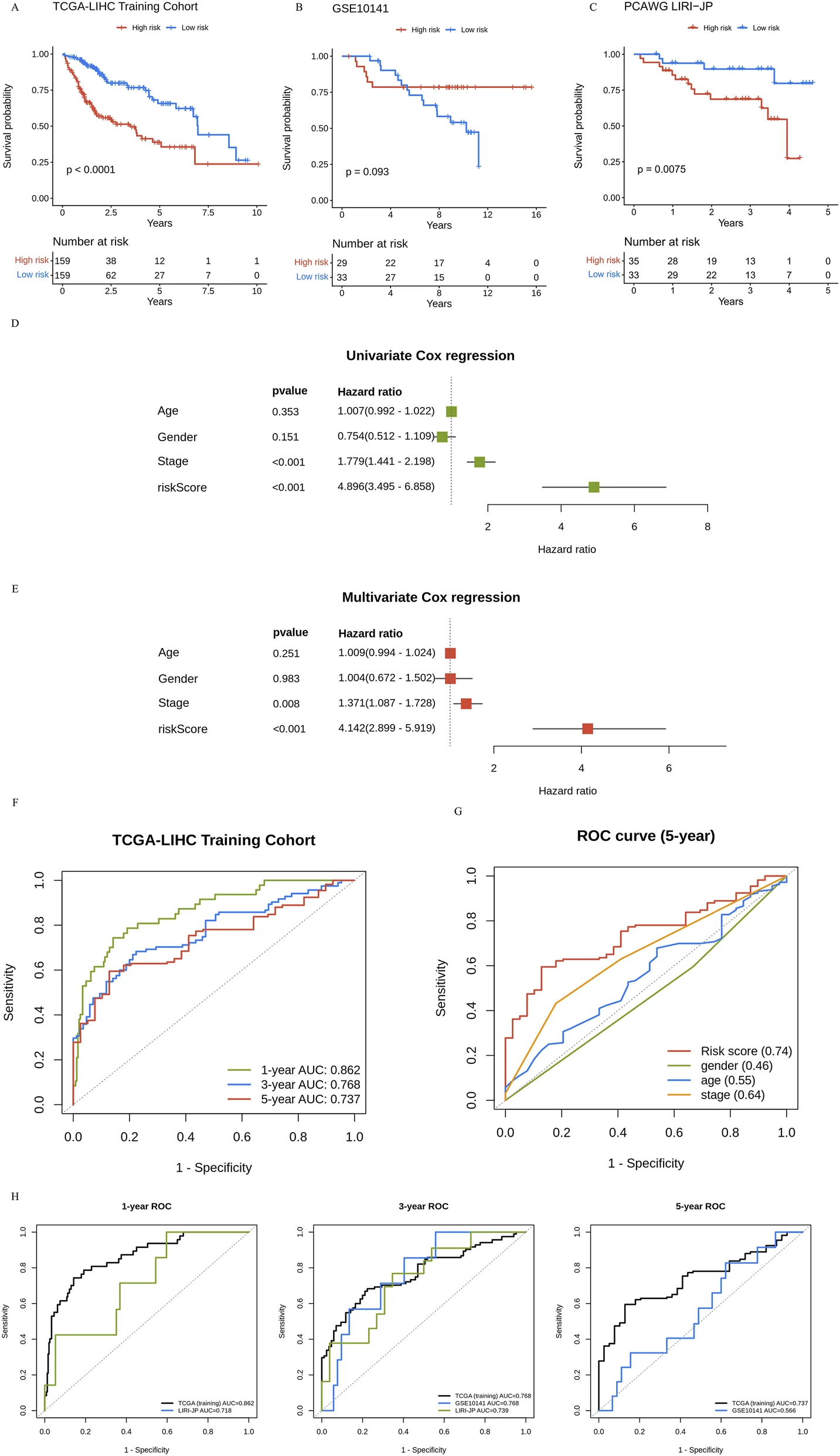
Prognostic stratification and predictive performance evaluation of the 20-gene prognostic risk model in HCC. **(A–C)** Kaplan–Meier overall survival curves of patients in the high- and low-risk groups in the TCGA-LIHC training cohort **(A)**, the GSE10141 external validation cohort **(B)**, and the LIRI-JP external validation cohort **(C)**, with between-group differences assessed by the log-rank test. **(D,E)** Univariate Cox regression analysis **(D)** and multivariate Cox regression analysis **(E)** of the risk score and clinical variables in the TCGA-LIHC training cohort, presented as HR and 95% confidence intervals. **(F)** Time-dependent ROC curves of the 20-gene risk model for predicting 1-, 3-, and 5-year overall survival in the TCGA-LIHC training cohort. **(G)** ROC curves of the risk score and clinical variables for predicting overall survival in the TCGA-LIHC training cohort. **(H)** Time-dependent ROC analysis of the risk model in the TCGA-LIHC training cohort and independent external validation cohorts. HR, hazard ratio; ROC, receiver operating characteristic; AUC, area under the curve.

The independent prognostic value of the risk score was further evaluated in the TCGA-LIHC training cohort. Univariate Cox regression analysis showed that the risk score was significantly associated with the overall survival of patients (HR = 4.90, 95% CI 3.50–6.86, *p* < 0.001, Figure 5D). After adjustment for age, sex, and stage, multivariate Cox regression confirmed that the risk score remained an independent prognostic factor (HR = 4.14, 95% CI 2.90–5.92, *p* < 0.001, Figure 5E). Time-dependent ROC analysis showed that the AUCs of the model for predicting 1-, 3-, and 5-year overall survival in the TCGA-LIHC training cohort were 0.862, 0.768, and 0.737, respectively (Figure 5F). At the 5-year prediction time point, the AUC of the risk score was 0.74, higher than that of clinical stage (0.64), age (0.55), and sex (0.46) (Figure 5G). Using the model coefficients and risk stratification threshold determined in the training cohort, the performance of the model was further evaluated in independent external cohorts. The C-index of the LIRI-JP cohort was 0.735 (95% CI: 0.615–0.854), and the difference in overall survival between the high- and low-risk groups was statistically significant (log-rank *P* = 0.0075); its 1- and 3-year time-dependent AUCs were 0.718 and 0.739, respectively, and the 5-year AUC was not estimated because the maximum follow-up duration was approximately 4.6 years. In contrast, the C-index of the GSE10141 cohort was 0.495 (95% CI: 0.358–0.632), and the difference in overall survival between the high- and low-risk groups did not reach statistical significance (log-rank *P* = 0.093); its 3- and 5-year time-dependent AUCs were 0.768 and 0.566, respectively (Figure 5H). These results suggest that the model has certain prognostic discriminative ability in the LIRI-JP cohort but unstable performance in the GSE10141 cohort.

To further assess the predictive performance of the risk model combined with clinical features, a nomogram integrating the risk score and key clinical variables was constructed based on the TCGA-LIHC training cohort (Figure 6A). Bootstrap calibration curves demonstrated favorable agreement between survival probabilities predicted by the nomogram and actual observed outcomes (Figure 6B). The clinical association heatmap showed that an elevated risk score was associated with a later stage (Figure 6C).

**FIGURE 6:**
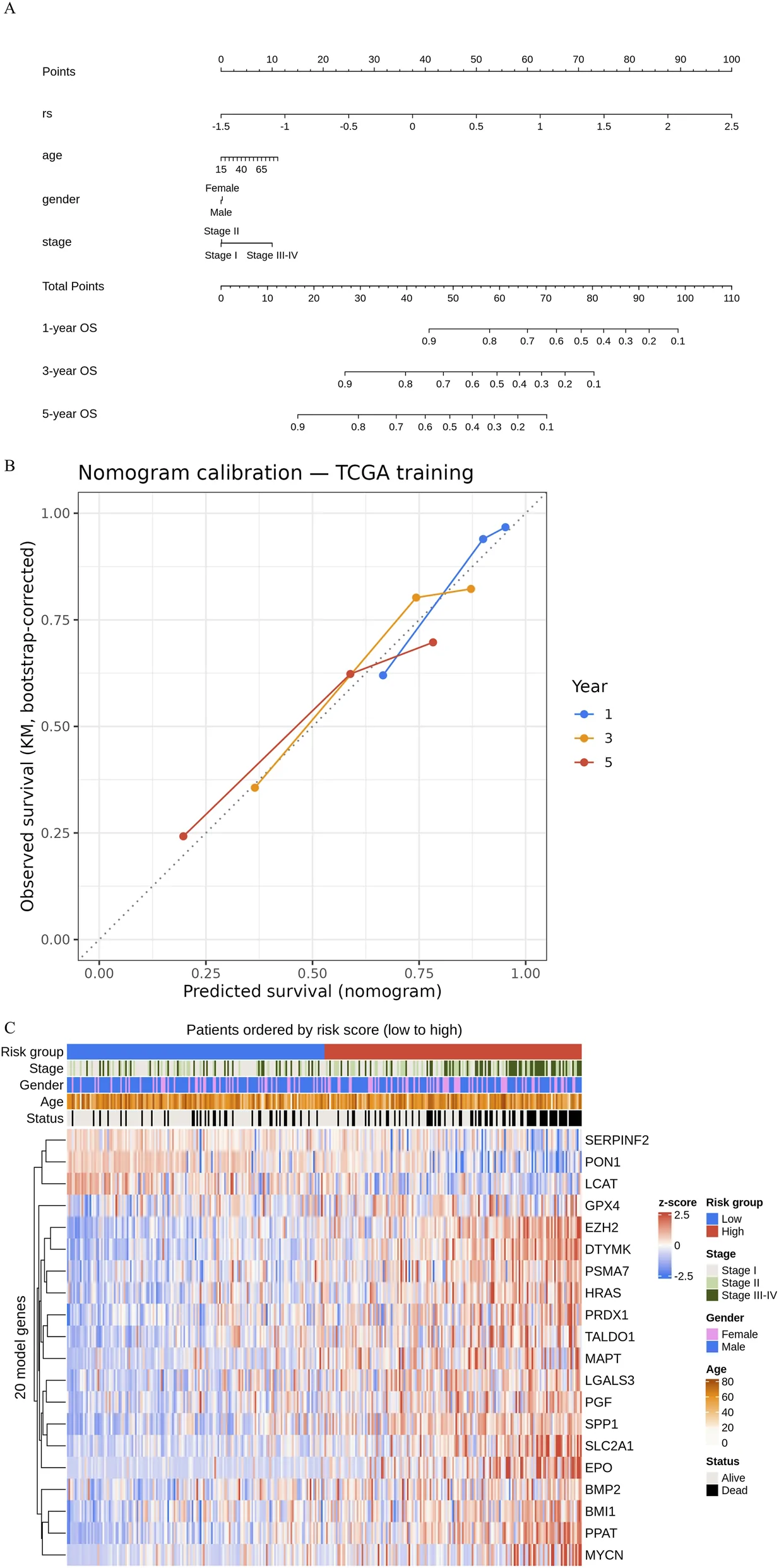
Prognostic nomogram constructed based on the TCGA-LIHC training cohort and its performance evaluation. **(A)** Nomogram integrating the 20-gene risk score and clinical variables for predicting overall survival. **(B)** Bootstrap-corrected calibration curves used to evaluate the agreement between the 1-, 3-, and 5-year overall survival probabilities predicted by the nomogram and the actual observed outcomes. **(C)** Heatmap of the association between the risk score and clinicopathological characteristics. Bootstrap, Bootstrap resampling.

Overall, the 20-gene prognostic model demonstrated certain prognostic discriminative ability in the TCGA-LIHC training cohort and the LIRI-JP external validation cohort, but its predictive performance in the GSE10141 cohort was limited, suggesting that the cross-platform applicability and long-term prognostic prediction ability of the model still need to be further validated in more independent cohorts.

### 3.5 Distinct immune cell infiltration patterns

To evaluate the tumor immune microenvironment characteristics of patients in different risk groups, CIBERSORT and ssGSEA were used to analyze the differences in immune cell infiltration and immune-related functions. CIBERSORT analysis showed that, among the 22 immune cell types, the estimated infiltration proportions of 8 cell types differed significantly between the high- and low-risk groups (FDR < 0.05). Specifically, the estimated infiltration proportions of M0 macrophages, activated CD4 memory T cells, follicular helper T cells (Tfh), resting dendritic cells, and regulatory T cells (Tregs) were higher in the high-risk group, whereas those of resting CD4 memory T cells, monocytes, and resting mast cells were higher in the low-risk group (Figure 7A). Further ssGSEA analysis showed that, among the 29 immune-related signatures, 10 differed significantly between the high- and low-risk groups (FDR < 0.05). Compared with the low-risk group, the high-risk group had higher scores related to macrophages, Tregs, activated dendritic cells (aDCs), and immature dendritic cells (iDCs), as well as higher MHC class I molecule-related and antigen-presenting cell co-stimulation (APC co-stimulation) scores, whereas the low-risk group had higher scores related to mast cells, NK cells, and type I and type II interferon responses (Figure 7B). These results suggest that the immune cell composition and immune-related functional characteristics differ between patients in different risk groups.

**FIGURE 7:**
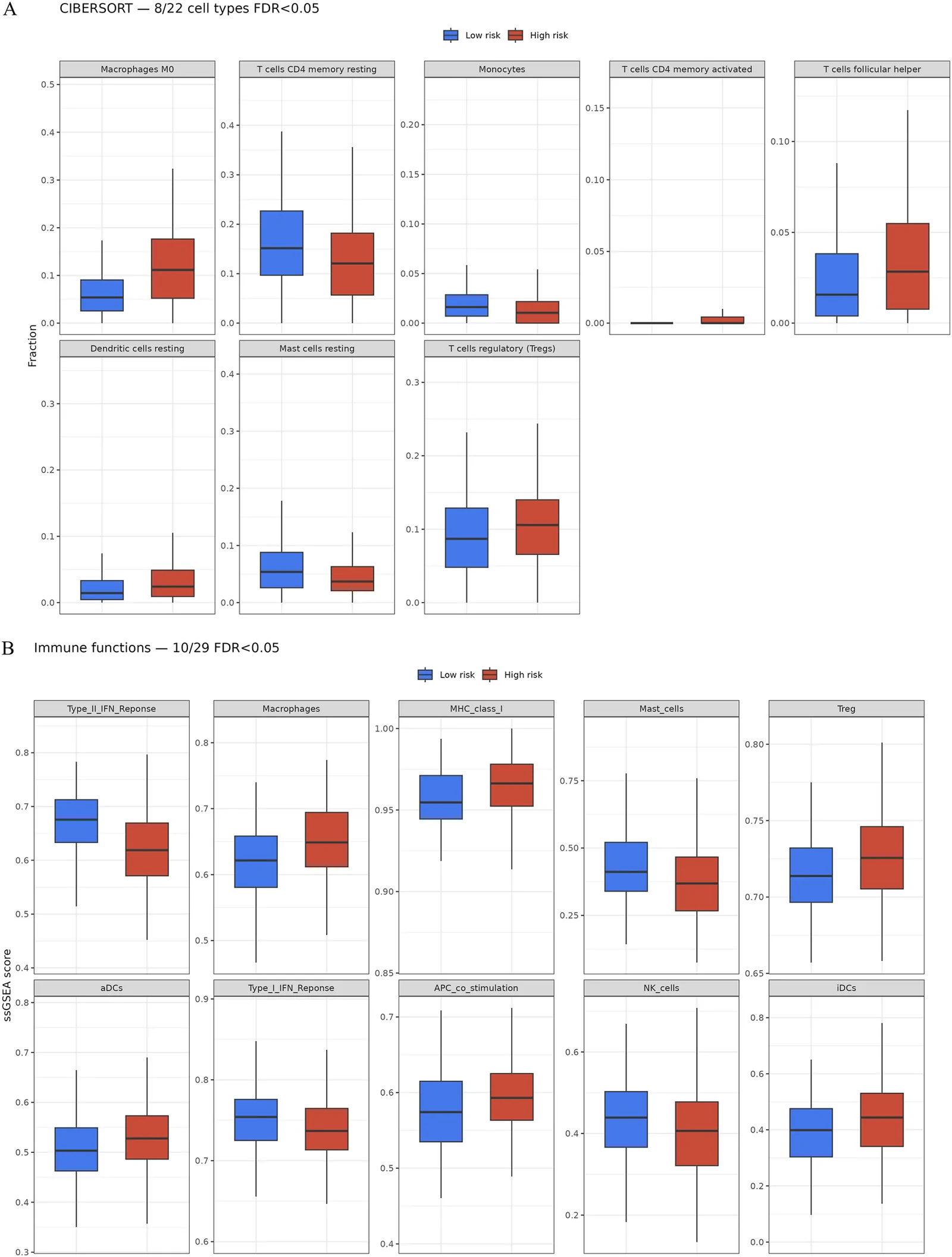
Immune cell composition and immune-related functional characteristics of patients in different risk groups. **(A)** CIBERSORT analysis of the estimated relative proportions of 22 immune cell types in the high- and low-risk groups, showing the 8 immune cell types with statistically significant between-group differences after multiple-testing correction. **(B)** ssGSEA evaluation of 29 immune-related signatures, showing the 10 immune function scores with significant differences between the high- and low-risk groups. Box plots represent the data distribution of the two groups, with blue representing the low-risk group and red representing the high-risk group. Between-group comparisons were performed using the Wilcoxon rank-sum test, with FDR < 0.05 as the significance screening criterion. CIBERSORT, cell-type identification by estimating relative subsets of RNA transcripts; ssGSEA, single-sample gene set enrichment analysis; FDR, false discovery rate.

### 3.6 Differences in immune characteristics and predicted drug sensitivity between patients in different risk groups

Immune subtype analysis showed that the distribution of immune subtypes differed significantly between patients in the high- and low-risk groups (χ^2^ test, *P* = 6.2 × 10^−7^). Compared with the low-risk group, the proportions of IFN-γ-dominant and wound-healing subtypes were relatively increased in the high-risk group, whereas the proportions of inflammatory and lymphocyte subtypes were relatively decreased (Figure 8A). Immunophenoscore (IPS) analysis showed that the overall IPS of the low-risk group was significantly higher than that of the high-risk group (Wilcoxon test, *P* = 2.0 × 10^−4^). In addition, the IPS-CTLA4, IPS-PD1, and IPS-PD1+CTLA4 scores were also significantly higher in the low-risk group (*P* = 0.0011, 0.0026, and 0.0041, respectively), suggesting differences in the predicted immune phenotypes between risk groups (Figure 8B). TIDE analysis showed that, compared with the low-risk group, the high-risk group had a lower overall TIDE score (*P* = 9.2 × 10^−4^) and T cell dysfunction score (*P* = 3.9 × 10^−4^) but a higher T cell exclusion score (*P* = 1.3 × 10^−5^) (Figure 8C). According to the TIDE predicted response classification, 135/159 patients (84.9%) in the high-risk group and 131/159 patients (82.4%) in the low-risk group were predicted to be responders to immune checkpoint inhibitor therapy, and the difference in the predicted response rate between the two groups was not statistically significant (Fisher’s exact test, *P* = 0.650; Supplementary Table S2). These results suggest that the immune-related predictive characteristics differ between patients in different risk groups, but it cannot be determined from these data whether there is a significant difference in the benefit of immune checkpoint inhibitor therapy.

**FIGURE 8:**
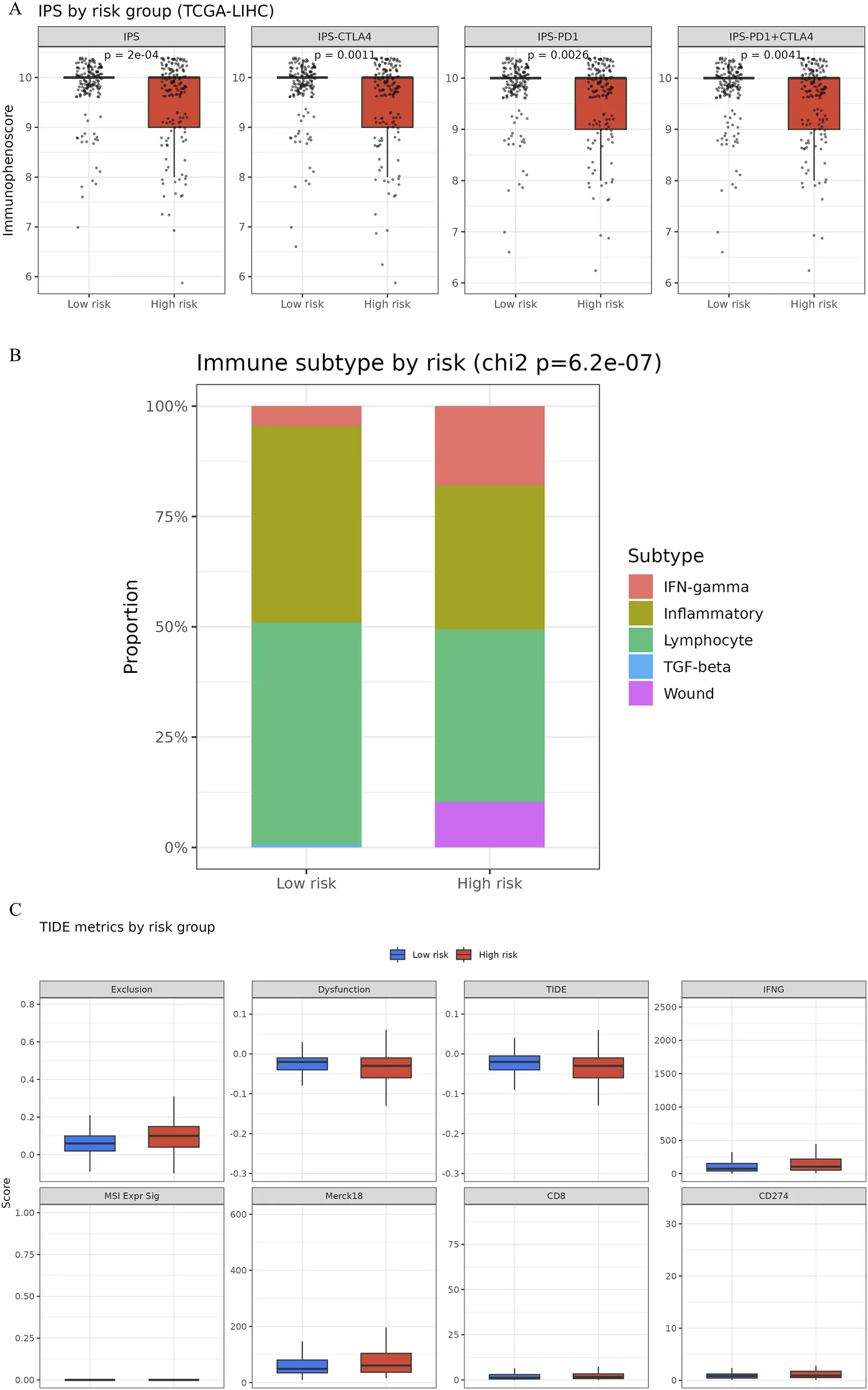
Immune subtype distribution and immunotherapy-related predictive characteristics of patients in different risk groups. **(A)** Immune subtype composition and proportional distribution of patients in the high- and low-risk groups, with between-group differences in immune subtype distribution compared using the χ2 test. **(B)** Comparison of the overall immunophenoscore (IPS) and the IPS-CTLA4, IPS-PD1, and IPS-PD1+CTLA4 scores between risk groups. **(C)** Comparison of the overall TIDE score, T cell dysfunction score, T cell exclusion score, and other TIDE-related immune indicators between risk groups. Between-group differences in IPS- and TIDE-related continuous variables were compared using the Wilcoxon rank-sum test. IPS, immunophenoscore; TIDE, Tumor Immune Dysfunction and Exclusion; PD1, programmed cell death protein 1; CTLA4, cytotoxic T-lymphocyte-associated protein 4.

Furthermore, drug sensitivity prediction analysis was performed using oncoPredict based on GDSC2 drug response data. The results showed that, among the 198 drugs included in the analysis, the predicted sensitivities of 123 drugs differed significantly between the high- and low-risk groups (FDR < 0.05), with 55 drugs showing higher predicted sensitivity in the high-risk group and 68 drugs showing higher predicted sensitivity in the low-risk group. Analysis of representative drugs showed that the high-risk group had lower predicted IC_50_ values for GDC0810, Taselisib, Dasatinib, BDP-00009066, and Fulvestrant, whereas the low-risk group had lower predicted IC_50_ values for PLX-4720, PF-4708671, JAK1_8709, SB505124, TAF1_5496, JQ1, and OF-1 (Figure 9). These results suggest that different risk groups may have distinct drug response characteristics, but these findings are based on computational predictions and still require further experimental and clinical validation.

**FIGURE 9:**
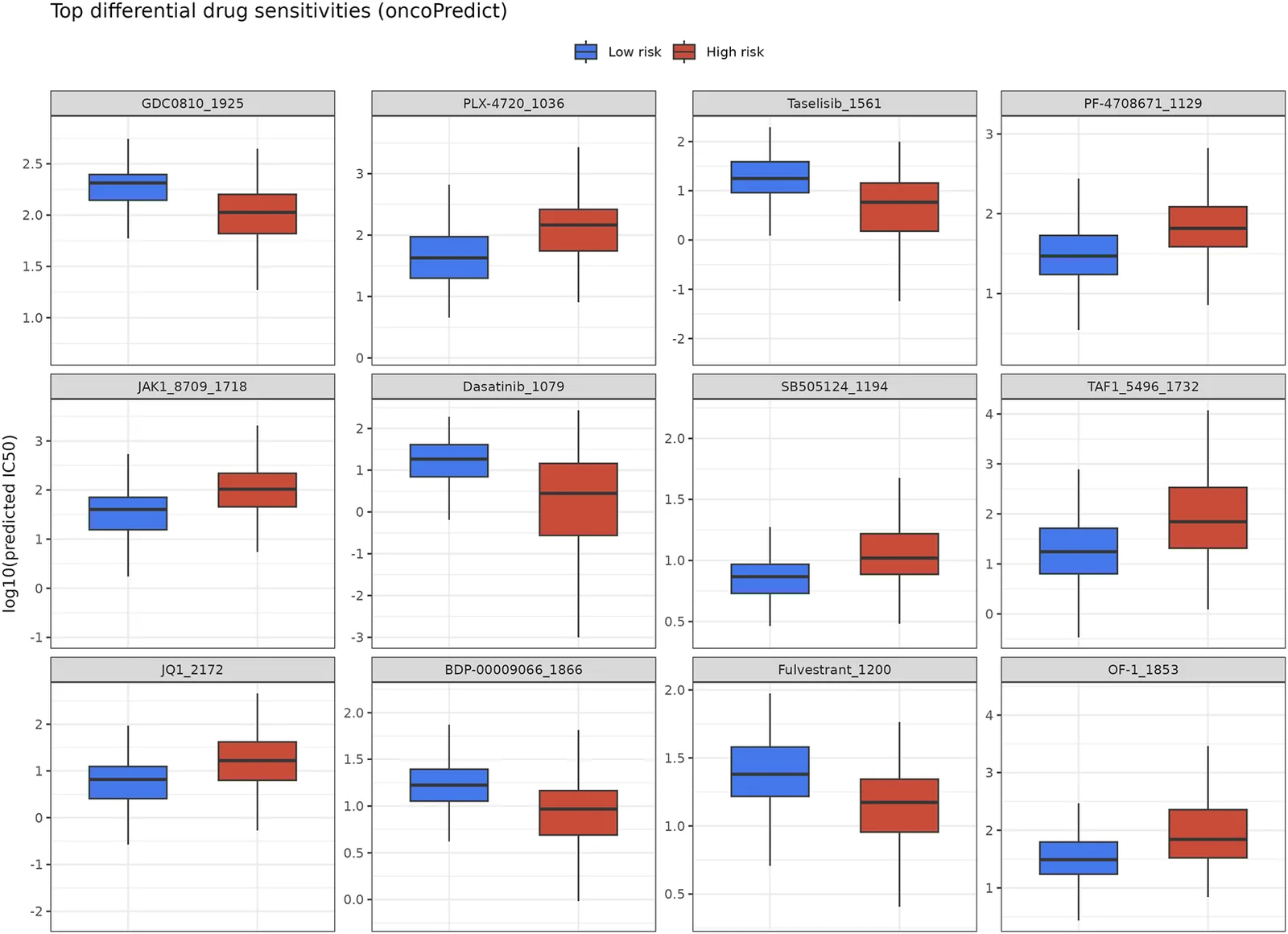
Differences in computationally predicted drug sensitivity between patients in different risk groups. Based on GDSC2 drug response data, oncoPredict was used to predict the potential sensitivity of TCGA-LIHC patients to different drugs, and 12 representative drugs with differential patterns between the high- and low-risk groups are shown. The vertical axis represents log10-transformed predicted IC_50_ values, with blue representing the low-risk group and red representing the high-risk group. Lower predicted IC_50_ values indicate higher computationally predicted drug sensitivity. GDSC, Genomics of Drug Sensitivity in Cancer; IC_50_, half-maximal inhibitory concentration.

### 3.7 Prognostic associations and expression characteristics of GSS and its relationship with the risk model

Within the pre-defined FAMGs set, differential expression analysis identified a total of 974 DE-FAMGs, including GSS, which encodes glutathione synthetase (Supplementary Table S1). In addition, univariate Cox regression analysis showed that GSS was significantly associated with the overall survival of HCC patients (HR = 1.369, 95% CI: 1.132–1.657, FDR = 0.0067), suggesting that GSS exhibits both disease-related expression changes and prognostic association characteristics (Supplementary Table S2). Further analysis revealed that GSS expression was significantly elevated in HCC tissues (n = 371) compared with normal liver tissues (n = 50) (log2FC = 0.81, *P* = 6.4 × 10^−20^; Supplementary Figure S2A). Kaplan–Meier survival analysis showed that patients with high GSS expression in the TCGA-LIHC cohort had significantly shorter overall survival than those with low expression (log-rank *P* = 2.2 × 10^−5^, HR = 2.23; Supplementary Figure S2B). In the independent LIRI-JP validation cohort, high GSS expression was likewise associated with poorer overall survival (log-rank *P* = 0.024; Supplementary Figure S2C). Further analysis of the relationship between GSS and the 20-gene prognostic model showed that the GSS expression level was significantly positively correlated with the model risk score (Spearman r = 0.47, *P* < 1 × 10^−15^; Supplementary Figure S2D). Gene expression correlation analysis showed that GSS was significantly positively correlated with PSMA7 (r = 0.59), DTYMK (r = 0.49), PRDX1 (r = 0.41), TALDO1 (r = 0.39), and GPX4 (r = 0.30) (all *P* < 1 × 10^−7^; Supplementary Figure S2E). In addition, the GSS expression level of patients in the high-risk group was significantly higher than that in the low-risk group (*P* = 3.8 × 10^−13^; Supplementary Figure S2F).

Despite its univariate prognostic relevance, GSS was not included in the final 20-gene prognostic model by Elastic Net Cox regression. When GSS was further added to the model, its regression coefficient did not reach statistical significance (Wald *P* = 0.568), the incremental improvement in model fit was not statistically significant (likelihood ratio test *P* = 0.567), and the C-index increased only from 0.767 to 0.768, indicating that GSS did not provide clear additional prognostic discrimination. Considering that GSS belongs to the pre-defined FAMGs set, was screened as a candidate gene in both the differential expression analysis and the univariate Cox regression analysis, exhibits cross-cohort prognostic associations and significant correlations with the model risk score and the expression of some model genes, and participates in glutathione biosynthesis and the regulation of cellular redox homeostasis, this study selected GSS as a candidate gene for functional validation outside the 20-gene prognostic model, and further explored its expression characteristics and potential biological role in HCC through single-cell transcriptome analysis and *in vitro* functional experiments.

### 3.8 GSS is highly expressed in malignant hepatocytes of HCC and correlates with poor prognosis

As the primary catalyst for glutathione synthesis, GSS is essential for maintaining cellular antioxidant capacity (Lapenna, 2023). The involvement of GSS in tumor development is context-dependent, with evidence supporting its capacity to both accelerate and inhibit cancer progression (Meng et al., 2025; Alwahsh et al., 2024). To further explore the expression profile and potential role of GSS in HCC, integrative validation was performed using scRNA-seq, survival analysis, and functional experiments.

After rigorous quality control and filtering of the GSE149614 dataset (Supplementary Figure S3), a total of 24 cell clusters were identified (Figure 10A). Based on canonical marker genes, these clusters were annotated into 12 cell types, including T cells, NK cells, Kupffer cells, hepatocytes, endothelial cells, plasma cells, DCs, fibroblasts, B cells, epithelial cells, and unknown-cell populations (Figures 10B,C).

**FIGURE 10:**
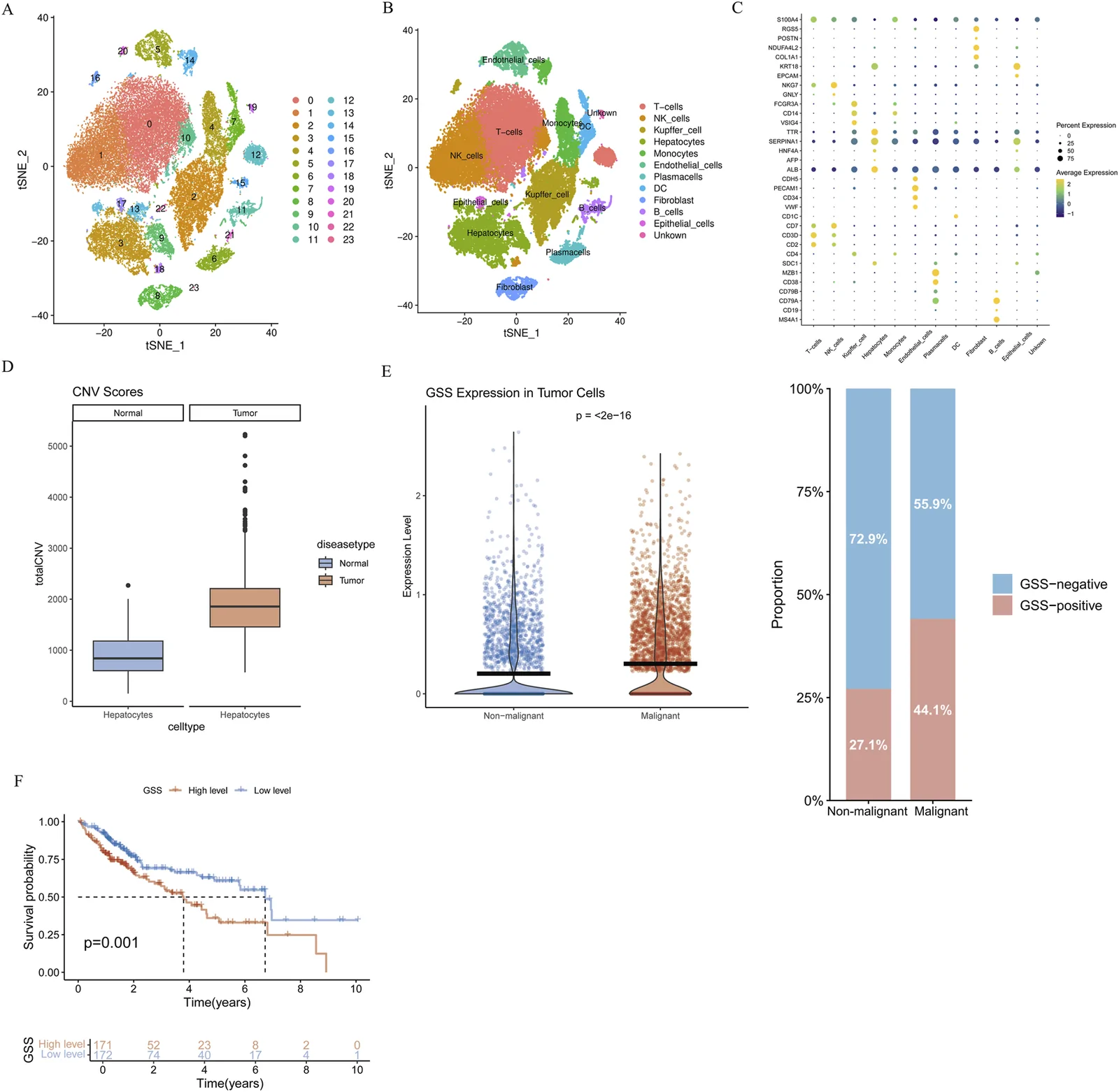
Expression characteristics of GSS in malignant hepatocytes of HCC and its prognostic associations. **(A)** t-SNE plot showing the distribution of different cell clusters in the single-cell transcriptome data. **(B)** t-SNE plot showing the distribution of annotated cell types. **(C)** Bubble plot showing the expression characteristics of canonical marker genes across cell types. **(D)** Comparison of hepatocyte CNV scores calculated based on inferCNV, used to assist in identifying putatively malignant and non-malignant hepatocytes. **(E)** Expression characteristics of GSS in putatively non-malignant and malignant hepatocytes; the left violin plot compares GSS expression levels between the 2 cell groups, and the right stacked bar plot shows the proportions of GSS-positive and GSS-negative cells in the two groups. **(F)** Kaplan–Meier overall survival curves of patients with high and low GSS expression in the TCGA-LIHC cohort. GSS, glutathione synthetase; HCC, hepatocellular carcinoma; CNV, copy number variation; t-SNE, t-distributed stochastic neighbor embedding.

inferCNV analysis was subsequently performed on hepatocytes to characterize GSS expression in malignant hepatocytes. Hepatocytes derived from normal tissues were set as the reference baseline to infer CNV, and unsupervised clustering was conducted according to CNV scores. Cells with high CNV scores exhibited significantly higher CNV levels than those with low CNV scores (Figure 10D), and were accordingly defined as malignant and non-malignant cells, respectively. Malignant cells were predominantly enriched in tumor samples, further supporting the reliability of this classification (Supplementary Figure S4). Compared with non-malignant hepatocytes, malignant hepatocytes displayed markedly elevated GSS expression (*p* < 0.0001). The proportion of GSS-positive cells increased from 27.1% to 44.1%, whereas the fraction of GSS-negative cells decreased from 72.9% to 55.9% (Figure 10E). In addition, high GSS expression was significantly associated with unfavorable overall survival (*p* = 0.001; Figure 10F). Collectively, these results demonstrate that GSS is upregulated in malignant hepatocytes of HCC and correlates with poor patient prognosis.

### 3.9 Functional investigation of GSS in HCC malignant progression

As an initial step toward understanding its role in HCC progression, GSS expression was first compared in normal and malignant hepatocytes (Hep3B, Huh-7, SNU-182), showing significant upregulation in HCC cells (*p* < 0.01) (Figures 11A,B). Subsequently, GSS knockdown cell models (si-NC, si-GSS) were established in Huh-7 cells with endogenous high GSS expression, while GSS overexpression cell models (oe-NC, oe-GSS) were constructed in SNU-182 cells with endogenous low GSS expression. qRT-PCR and Western blot analyses confirmed efficient knockdown and overexpression of GSS (*p* < 0.001; Figures 11C,D), which met the requirements for subsequent functional validation experiments. Both CCK-8 and EdU proliferation assays revealed significantly impaired proliferative capacity of Huh-7 cells upon GSS knockdown (*p* < 0.0001) (Figures 11E,F). Transwell migration and invasion assays indicated significantly reduced migratory and invasive abilities of si-GSS cells (*p* < 0.001) (Figure 11G). Conversely, overexpression of GSS exerted the opposite effects (Figure 11E–G). Collectively, GSS is highly expressed in HCC and significantly promotes the proliferation, migration, and invasion of HCC cells *in vitro*, supporting its essential function in sustaining HCC malignancy.

**FIGURE 11:**
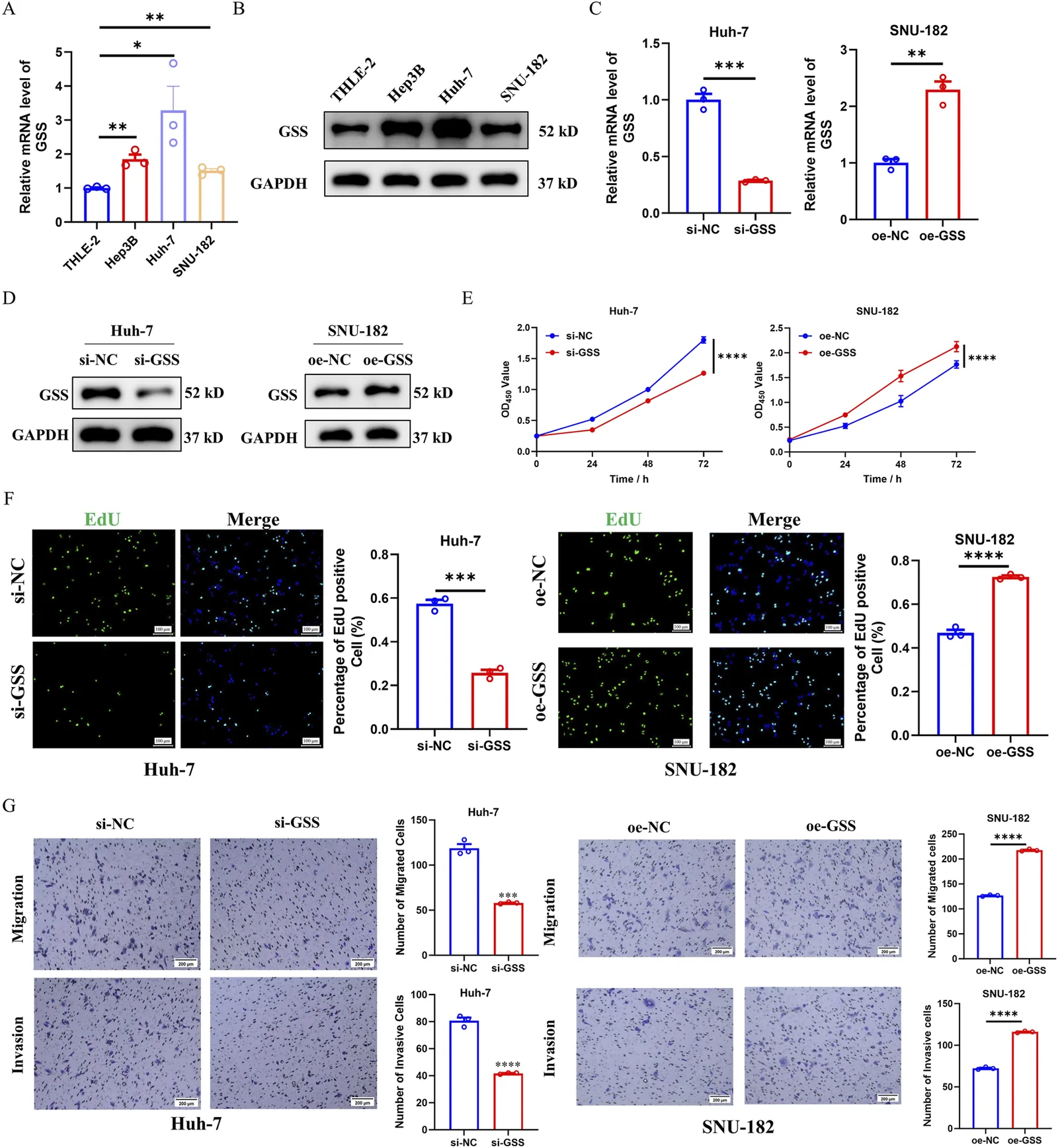
Effects of GSS on the proliferation, migration, and invasion phenotypes of HCC cells. **(A)** qRT-PCR detection of GSS mRNA expression levels in the normal hepatic epithelial cell line THLE-2 and the HCC cell lines Hep3B, Huh-7, and SNU-182. **(B)** Western blot detection of GSS protein expression levels in THLE-2, Hep3B, Huh-7, and SNU-182 cells. **(C)** qRT-PCR verification of the transfection efficiency of GSS knockdown in Huh-7 cells and GSS overexpression in SNU-182 cells. **(D)** Western blot verification of the protein expression changes of GSS knockdown in Huh-7 cells and GSS overexpression in SNU-182 cells. **(E)** CCK-8 assay evaluating the effects of GSS knockdown or overexpression on the viability/proliferation-related phenotypes of Huh-7 and SNU-182 cells. **(F)** EdU incorporation assay evaluating the effects of GSS knockdown or overexpression on the proliferative capacity of Huh-7 and SNU-182 cells. **(G)** Transwell assays evaluating the effects of GSS knockdown or overexpression on the migratory and invasive capacities of Huh-7 and SNU-182 cells. All quantitative experiments were performed with three independent biological replicates (n = 3). Data are presented as mean ± SEM. *P < 0.05, ***P < 0.001, ****P < 0.0001. GSS, glutathione synthetase; HCC, hepatocellular carcinoma; qRT-PCR, quantitative reverse transcription polymerase chain reaction; WB, Western blot; EdU, 5-ethynyl-2′-deoxyuridine.

## 4 Discussion

HCC exhibits prominent molecular and metabolic heterogeneity. Metabolic reprogramming not only modulates tumor-cell survival and proliferation but is also tightly linked to the tumor immune microenvironment and patient prognosis. Previous studies have uncovered molecular heterogeneity in HCC from the perspectives of metabolic gene classification, immune status, and oxidative stress, and have constructed corresponding prognostic signatures (Cui et al., 2023; Gu et al., 2024). Transcriptomic investigations have also demonstrated that HCC progression is accompanied by extensive molecular alterations and remodeling of the tumor immune ecosystem (Okrah et al., 2018). On this basis, the present study focused on FAMGs and established a 20-gene prognostic risk model for HCC using Elastic Net Cox regression, and evaluated its predictive performance and model interpretability through Bootstrap resampling, independent external cohorts, and SHAP analysis. Meanwhile, by integrating immune characteristics, predicted drug sensitivity, single-cell transcriptomics, and *in vitro* functional experiments, the study was conducted at two levels: prognostic stratification and candidate gene function. The results provide further evidence for understanding the relationships between FAMGs-related molecular signatures and the prognostic heterogeneity and malignant phenotype of HCC.

FAM constitutes a core component of the one-carbon metabolic network and is closely involved in nucleotide synthesis, methyl-donor production, DNA replication, and redox homeostasis. Existing evidence indicates that chromosomal instability promotes hepatocarcinogenesis (Begus-Nahrmann et al., 2012). Fluctuations in folate levels modulate oncogene-induced replication stress, DNA damage, and tumor-forming capacity by regulating nucleotide supply (Lamm et al., 2015). In this study, functional enrichment analysis of differentially expressed FAMGs showed that these genes were mainly involved in DNA replication and repair, the cell cycle, stress response, and metabolism-related processes, and were enriched in pathways such as folate-mediated one-carbon metabolism, suggesting that the broadly defined FAMGs set may be associated with multiple biological processes in HCC. Further GO biological process analysis of the 20 prognostic model genes revealed that they were mainly enriched in terms such as cellular response to chemical stress, positive regulation of MAPK and ERK1/ERK2 signaling cascades, regulation of kinase activity, and regulation of miRNA metabolic process. These results suggest that the constructed prognostic signature may reflect molecular states related to cellular stress and signaling regulation. It should be noted that the model genes in this study were derived from a broadly defined FAMGs candidate set, and the functional enrichment results cannot prove that all model genes are directly involved in classical FAM. Therefore, the model is more appropriately interpreted as a prognostic molecular signature constructed from a FAMGs candidate set, and its mechanistic links with specific folate metabolic processes still require further validation.

The 20-gene signature constructed in this study showed prognostic stratification in the TCGA-LIHC training cohort and the LIRI-JP external validation cohort, while its performance was limited in GSE10141. In the TCGA-LIHC training cohort, the risk score remained independently associated with overall survival after adjustment for clinical covariates. Prior studies have built HCC prognostic models based on m^6^A-related lncRNAs, ceRNA networks, and other molecular biomarkers (Xu and Liu, 2022; Zhang et al., 2022; Zhang et al., 2019), illustrating that molecular signatures can complement conventional clinical indicators from distinct biological dimensions. In this study, a 20-gene prognostic model was further constructed from FAM-related molecular signatures using Elastic Net Cox regression, and the contribution of different genes to model prediction was evaluated by SHAP analysis. The C-index of the model in the TCGA-LIHC training cohort was 0.767, similar to the apparent discriminative performance of the LASSO Cox model. However, after correction by 999 Bootstrap resampling iterations, the C-index of the model decreased to 0.676, suggesting a certain degree of optimism in the apparent predictive performance in the training cohort. In addition, the training cohort contained 108 death events, and the events per variable (EPV) of the final 20-gene model was 5.4, indicating that attention should still be paid to model complexity and the potential risk of overfitting. Moreover, the 231 candidate genes identified before penalized regression corresponded to only 0.47 events per candidate variable, further highlighting the potential instability introduced during the variable-screening stage. Although the complete model-selection procedure was repeated within each Bootstrap resample to account for selection-related optimism, these findings indicate that the model requires further independent validation before clinical application. In independent external validation, the model showed certain prognostic discriminative ability in the LIRI-JP cohort (C-index = 0.735) but limited predictive performance in the GSE10141 cohort (C-index = 0.495), and the difference in overall survival between the high- and low-risk groups did not reach statistical significance. GSE10141 used a DASL gene expression profiling platform based on FFPE samples, whereas both LIRI-JP and TCGA-LIHC used RNA-seq technology; differences in detection platforms and sample processing may affect the cross-cohort predictive performance of the model, but their specific impact still requires further investigation. Therefore, the 20-gene prognostic signature constructed in this study has certain potential for prognostic stratification, but its predictive stability, cross-platform generalizability, and clinical application value still need to be further validated in larger-scale, multicenter independent cohorts.

Based on the prognostic model analysis, this study further focused on the potential biological significance of GSS in HCC. GSS encodes glutathione synthetase, a key enzyme in the biosynthesis of glutathione (GSH), which plays an important role in maintaining cellular redox homeostasis and responding to oxidative stress. Previous studies have shown that, in specific tumor contexts, hypoxia-related metabolic reprogramming can promote the adaptation of tumor cells to metabolic and oxidative stress by enhancing GSS-mediated GSH synthesis, thereby supporting tumor growth (Lin et al., 2024). In the present study, GSS was derived from the pre-defined FAMGs set and simultaneously exhibited differential expression and prognostic association characteristics: GSS expression was elevated in HCC tissues, and its high expression was associated with poorer overall survival in both the TCGA-LIHC and LIRI-JP cohorts; in addition, GSS expression was positively correlated with the risk score of the 20-gene model and the expression of some model genes. These results suggest that GSS may be associated with poor prognosis and high-risk molecular characteristics of HCC. Notably, GSS was not included in the final 20-gene model by Elastic Net Cox regression, and adding it to the model did not yield significant incremental predictive value, indicating that the prognostic association of GSS is not equivalent to an independent predictive contribution to the model. Therefore, rather than treating GSS as a core component of the 20-gene prognostic model, this study conducted further functional research on it as an independent candidate gene based on its differential expression, cross-cohort prognostic associations, and glutathione metabolism-related biological functions. Single-cell analysis showed that both the expression level and the proportion of positive cells of GSS were elevated in hepatocytes inferred to be malignant; further *in vitro* experiments showed that knockdown of GSS inhibited the proliferation, migration, and invasion of HCC cells, whereas overexpression of GSS produced the opposite effects. These results, from different levels including tissue expression, patient prognosis, single-cell expression, and cell function, support the possible involvement of GSS in maintaining the malignant phenotype of HCC and suggest that its potential role may be related to the redox metabolic adaptation of tumor cells. However, this study has not directly measured GSH levels, ROS status, or related metabolic flux, nor has it conducted corresponding mechanistic rescue experiments; therefore, it currently remains unclear whether the promotion of the malignant phenotype of HCC by GSS is mediated by GSH-dependent redox regulation, and its specific molecular mechanisms still require further investigation.

Beyond intrinsic tumor-cell metabolic traits, divergent immune-microenvironment characteristics were observed between risk subgroups. HCC progression is accompanied by substantial remodeling of the immune ecosystem (Okrah et al., 2018), and metabolism- and immune-related molecular signatures are closely associated with the HCC immune microenvironment (Cui et al., 2023; Gu et al., 2024). High-risk status in our cohort was accompanied by compositional shifts in myeloid and lymphoid compartments as well as alterations in multiple immune functions, suggesting that FAM-related molecular status may not only reflect intrinsic tumor metabolism but also correlate with the tumor immune ecosystem. These observations are consistent with earlier reports that metabolic profiles and immune landscapes jointly determine HCC prognosis, implying potential crosstalk between FAM-associated molecular features and immune-ecosystem remodeling. It should be emphasized that CIBERSORT estimates relative immune-cell fractions inferred from bulk transcriptomic data rather than absolute cell abundance within tissues. Consequently, these results should be interpreted as differences in immune-cell composition across risk groups and cannot prove alterations in absolute immune-cell infiltration.

With advances in immune-checkpoint inhibitors and combinatorial regimens, immunotherapy has become an indispensable component of systemic treatment for advanced HCC (Marquardt et al., 2019; Liu Z. L. et al., 2021; Leone et al., 2021). However, the immune microenvironment of HCC exhibits marked heterogeneity, and it remains of great significance to identify molecular signatures that can reflect the immune status of patients and help identify populations potentially benefiting from treatment. The IPS and TIDE analyses in this study jointly indicate that FAM-related risk stratification is associated with immunotherapy-related predictive features to some extent, but the prediction patterns presented by the two methods are not entirely consistent. One possible explanation for this phenomenon is that IPS mainly integrates the expression characteristics of effector cells, immunosuppressive cells, immune checkpoints, and immunoregulatory molecules, whereas TIDE focuses on immune evasion mechanisms such as T cell dysfunction and T cell exclusion; therefore, the two reflect different dimensions of the tumor immune status. Notably, further TIDE predicted response classification did not show a significant difference between the high- and low-risk groups, suggesting that the differences in immune-related scores between risk groups cannot simply be equated with differences in sensitivity to immune checkpoint inhibitor therapy. Therefore, at the current stage, it is more appropriate to interpret these results as an association between FAM-related risk stratification and different immune states, rather than to consider that this risk model can directly predict benefit from immunotherapy. In the future, its potential therapeutic predictive value still needs to be validated in independent HCC cohorts receiving immune checkpoint inhibitor therapy with objective efficacy and survival outcomes.

Owing to pronounced molecular heterogeneity in HCC, patients display highly variable sensitivity to systemic therapies. Identifying populations likely to benefit from treatment using molecular signatures is critical for precision oncology (Marquardt et al., 2019; Marks and Yee, 2016; Yu and Ma, 2024; Duan et al., 2019). Our drug-sensitivity prediction expands the potential implications of FAM-based risk stratification, suggesting that this signature captures both prognostic heterogeneity and therapeutic vulnerability in HCC and providing novel clues for interpreting inter-patient variation in drug responses from a metabolic perspective. As targeted therapies and combination strategies evolve (Liu Z. L. et al., 2021; Leone et al., 2021), further dissection of links between FAM-related molecular profiles and therapeutic sensitivity may facilitate candidate-drug screening and patient stratification. Since current drug-sensitivity evidence originates primarily from computational predictions, these findings require verification via pharmacological assays and clinical treatment cohorts.

This study has several limitations. First, this study was mainly based on retrospective public databases, and model development relied on a single TCGA-LIHC training cohort. Although Elastic Net Cox regression was used for variable selection and model performance was evaluated through 999 Bootstrap resampling iterations, the corrected C-index decreased from 0.767 to 0.676, suggesting a certain degree of optimism in the apparent predictive performance in the training cohort; meanwhile, the final model contained 20 genes while the training cohort had only 108 death events, and the model still carries risks of overfitting and unstable variable selection. Second, the external validation results showed obvious heterogeneity: the model had certain discriminative ability in the LIRI-JP cohort but limited predictive performance in the GSE10141 cohort. Differences in detection platforms, sample processing methods, patient composition, and the number of events across cohorts may affect model performance, and its stability and generalizability still need to be validated in the future in larger-scale, multicenter independent cohorts with different detection platforms. Third, the immune cell infiltration, immunotherapy response, and drug sensitivity analyses were mainly based on computational inference from transcriptomic data, lacking tissue-level validation, real-world treatment outcomes, and drug intervention experiments; therefore, the actual benefit of immunotherapy or drug therapy for patients cannot be determined from these data. Finally, although single-cell analysis and *in vitro* functional experiments support the association between GSS and the malignant phenotype of HCC, GSS was not included in the final prognostic model, and its functional research results cannot directly prove the biological mechanism of the 20-gene model. In addition, this study has not measured GSH, ROS, or related metabolic flux, nor has it verified the specific molecular pathways of GSS action through mechanistic rescue experiments; therefore, its potential metabolic regulatory mechanisms still require further investigation.

## 5 Summary

In summary, this study constructed a 20-gene prognostic risk model for HCC based on FAMGs, and evaluated its predictive performance and model interpretability through Bootstrap internal validation, independent external cohorts, and SHAP analysis. The model demonstrated certain prognostic discriminative ability in the TCGA-LIHC training cohort and the LIRI-JP external validation cohort, but its performance declined after Bootstrap correction and its predictive performance in the GSE10141 cohort was limited, suggesting that the stability and cross-platform generalizability of the model still require further validation. In addition, different risk groups exhibited distinct immune-related characteristics and computationally predicted drug sensitivities, but their clinical value for treatment prediction has not yet been confirmed. As a candidate gene for functional research beyond the model, the expression and prognostic association characteristics of GSS in HCC, together with the single-cell and *in vitro* experimental results, support its possible involvement in maintaining the malignant phenotype of HCC. This study provides a basis for prognostic stratification of HCC and further research on related metabolic candidate molecules.

## Data Availability

Publicly available datasets were analyzed in this study. This data can be found here: The dataset used and analyzed during this study has been fully stored on Zenodo and can be accessed via https://doi.org/10.5281/zenodo.22092126.

## Supplementary material

SUPPLEMENTARY FIGURE S1 Comparison of variable selection methods for the prognostic model and GO biological process enrichment analysis of model genes. **(A)** Comparison of the model genes selected by Elastic Net Cox and LASSO Cox regression. **(B)** GO biological process (BP) enrichment analysis of the 20 prognostic model genes. GO, Gene Ontology; BP, biological process; LASSO, least absolute shrinkage and selection operator.

SUPPLEMENTARY FIGURE S2 Expression and prognostic characteristics of GSS in HCC and its relationship with the 20-gene risk model. **(A)** Comparison of GSS expression levels between HCC tissues (n = 371) and normal liver tissues (n = 50) in the TCGA-LIHC cohort, with between-group differences assessed by the Wilcoxon rank-sum test. **(B)** Kaplan–Meier overall survival curves of patients with high and low GSS expression in the TCGA-LIHC cohort, with between-group differences assessed by the log-rank test. **(C)** Kaplan–Meier overall survival curves of patients with high and low GSS expression in the independent LIRI-JP validation cohort. **(D)** Spearman correlation analysis evaluating the correlation between GSS expression level and the risk score of the 20-gene model. **(E)** Spearman correlation analysis evaluating the correlations between GSS and the expression levels of the model genes PSMA7, DTYMK, PRDX1, TALDO1, and GPX4. **(F)** Comparison of GSS expression levels between patients in the high- and low-risk groups of the 20-gene model, with between-group differences assessed by the Wilcoxon rank-sum test. GSS, glutathione synthetase; HCC, hepatocellular carcinoma; HR, hazard ratio; r, Spearman correlation coefficient; FDR, false discovery rate.

SUPPLEMENTARY FIGURE S3 Quality control of single-cell RNA sequencing data and evaluation of dimensionality reduction parameters. **(A)** Distribution of the number of detected genes per cell (nFeature_RNA), total UMI counts (nCount_RNA), and the proportion of mitochondrial gene expression in single-cell data before quality control. **(B)** Distribution of the above indicators after quality control. **(C)** Results of highly variable gene screening, showing 2,000 highly variable genes. **(D)** PCA ElbowPlot used to evaluate the variance contribution of principal components. **(E)** JackStraw analysis used to evaluate the statistical significance of principal components. PCA, principal component analysis; UMI, unique molecular identifier.

SUPPLEMENTARY FIGURE S4 Identification of malignant hepatocytes in HCC based on inferCNV analysis. The copy number variation (CNV) status of hepatocytes was inferred based on inferCNV, and cells were classified according to CNV scores. The figure shows the CNV score distribution, cell composition, and between-group differences in CNV scores of different cell populations, and compares the distribution characteristics of putatively malignant and non-malignant hepatocytes in normal and HCC tissues, so as to assist in evaluating the rationality of the malignant cell identification results. CNV, copy number variation; HCC, hepatocellular carcinoma.

SUPPLEMENTARY TABLE S1 Differentially expressed folate metabolism-related genes between HCC and normal liver tissues.

SUPPLEMENTARY TABLE S2 Univariate Cox regression analysis of prognosis-associated folate metabolism-related genes in the TCGA-LIHC cohort.
